# Manual Laryngeal Therapy Across Clinical Populations: A Scoping Review

**DOI:** 10.3390/jcm15218671

**Published:** 2026-11-06

**Authors:** Ben Barsties von Latoszek, Zoë Thijs, Paula-Marie Dammer, Melina Maria Ippers

**Affiliations:** 1 School of Health, Education and Social Sciences, SRH University of Applied Sciences Heidelberg, 40215 Düsseldorf, Germany; 2 Department of Communication Disorders and Occupational Therapy, University of Arkansas, Fayetteville, AR 72701, USA; zthijs@uark.edu; 3 Faculty of Social- and Health Care Sciences, Ostbayerische Technische Hochschule, 93053 Regensburg, Germany; 4 Faculty of Social Work and Health, HAWK University of Applied Sciences and Arts Hildesheim/Holzminden/Göttingen, 31134 Hildesheim, Germany

**Keywords:** manual laryngeal therapy, manual circumlaryngeal therapy, laryngeal manual therapy, dysphonia, voice disorders, voice therapy, laryngeal discomfort

## Abstract

**Background/Objectives**: Manual laryngeal therapy is used in conservative behavioral management and encompasses hands-on techniques to address functional manifestations of dysphonia, dysphagia, and laryngeal discomfort. However, evidence regarding clinical indications, treatment protocols, and outcomes remains heterogeneous. This review mapped the available evidence on manual laryngeal therapy approaches, treated populations, outcome measures, and reported treatment effects. **Methods**: Following PRISMA-ScR guidelines, 6 databases were searched from inception to 26 August 2026, as well as grey literature. Primary studies and meta-analyses were charted separately to distinguish evidence levels and avoid double-counting. Study characteristics, clinical populations, therapeutic approaches, outcome measures, and reported treatment effects were extracted and synthesized descriptively. **Results**: The review identified 41 eligible publications comprising 4 meta-analyses and 37 primary studies, which comprised randomized controlled trials (*n* = 12), cohort studies (*n* = 5), case series (*n* = 12), and case studies (*n* = 8). Evidence concerned voice disorders, with Laryngeal Manual Therapy (LMT; 29.8%) and Manual Circumlaryngeal Therapy (MCT; 27.0%) representing the most frequently investigated approaches, followed by Voice Massage and Cricothyroid Visor Maneuver (each 8.1%), Larynx Manipulation and Vanderbilt Manual Intervention (each 5.4%), and other single evaluated manual techniques/approaches (16.2%). Treatment protocols and outcome measures varied substantially. Significant positive effects were reported for 53% of statistically assessable LMT outcome parameters and 62% of MCT. Meta-analyses consistently reported significant improvements in voice quality parameters following MCT, alongside benefits in selected voice function and articulation measures. Conversely, a meta-analysis investigating LMT found no significant improvements in perceived voice quality or self-evaluation compared with voice exercises or transcutaneous electrical nerve stimulation. **Conclusions**: Manual laryngeal therapy has mostly been investigated in functional voice disorders. Evidence suggests combined manual techniques with voice exercises to benefit voice outcomes, while passive treatment may better address laryngeal discomfort and related symptoms. Standardized protocols, diagnosis-specific outcome measures, and controlled studies are needed to establish clinical effectiveness.

## 1. Introduction

In conservative behavioral management, manual laryngeal therapy approaches used by therapists have attracted considerable interest in the field of patient care and research [1,2,3,4,5,6,7]. Manual laryngeal therapy can be classified as component of symptomatic therapy concept, an approach directed toward the modification of perceptual and functional manifestations of dysphonia, dysphagia, and vocal fold discomfort identified during comprehensive voice assessment. These approaches typically involve the manipulation of the structures and musculature in the laryngeal and paralaryngeal regions by a trained therapist or the client themselves using hands and fingers, often during phonation [4]. Despite this common ground between manual laryngeal therapies, multiple therapeutic approaches have been described in the literature, for example, by Aronson [8], Lieberman et al. [9,10], Mathieson et al. [11], and Roy [12].

An important target of such interventions is hyperfunctional laryngeal muscle activity or absence of laryngeal mobility. Manual treatment techniques are designed to reduce musculoskeletal tension in the (para)laryngeal muscles, which may contribute to vocal hyperfunction, and reduce excessive tension in the muscles surrounding the larynx [12,13]. Within this therapeutic framework, clinicians employ hands-on intervention strategies to facilitate the identification, establishment, and maintenance of more efficient and physiologically favorable patterns of voice and swallowing performance. The resulting changes in muscular tension may be associated with improvements in vocal pitch, breathiness, roughness, swallowing feasibility/safety, and sensations of discomfort or pain within the vocal tract. These characteristics can be assessed using a range of instrumental and non-instrumental methods [14,15], such as laryngeal endoscopy, surface electromyography, acoustic measures, X-ray-based techniques, and palpation methods. Palpation is the only non-instrumental method in this list and plays a key role in the tactile-perceptual assessment of laryngeal and extralaryngeal muscle tension. While some of these approaches have shown limited reliability and validity [16], more recent palpation-based assessment procedures, however, have demonstrated promising, and in some cases high, levels of validity and reliability including the ability to differentiate individuals, for example, with and without voice disorders in populations in which muscular hyperfunction may play a relevant role [17,18,19]. Key parameters of palpation-based assessments include the thyrohyoid space; the lateral, caudal, and cranial mobility of the larynx; and the cricothyroid space, assessed both at rest and during phonation [19]. In addition, a palpation assessment instrument should provide quantifiable measures and clearly defined patterns that allow for the reliable classification of the severity of muscular tension or hyperfunction in clinical practice [19].

The current evidence remains unclear regarding the populations treated with manual laryngeal therapy, the protocols implemented, and the outcome measures used to assess treatment effects. Consequently, the aim of this scoping review is to map the existing evidence landscape on the various approaches to manual laryngeal therapy. Specifically, we aim to describe the populations and clinical indications in which manual laryngeal therapy has been applied, the different manual approaches used, and the outcome measures and treatment effects reported in the literature.

## 2. Materials and Methods

### 2.1. Data Sources and Searches

This scoping review was conducted following the PRISMA-ScR (Preferred Reporting Items for Systematic Reviews and Meta-Analyses extension for Scoping Reviews) checklist [20] (Table S1) and was registered in the Open Science Framework before the start of screening. The primary research question was based on the PCC (population, concept, and context) format: What evidence is available regarding the use of manual laryngeal therapy, including populations and diagnoses in which it has been used, clinical indications, and the reported outcome measures, (concept) among populations with or at risk for laryngeal disorders (population) in clinical settings (context)? A systematic search of six databases (MEDLINE, CINAHL, PsychINFO, Embase, Web of Science, Ovid Emcare) was conducted without language or time restrictions from inception to August 26, 2026. In addition, a grey literature search of Google Scholar was used for the first 100 references. For the search, we used the terms “voice” and “musculoskeletal manipulations” along with relevant text words and translated this search string into the each database’s native vocabulary. Detailed search strategies were developed for each of the six databases (Table S2). In addition to searching the above databases, a hand-search was completed to identify potentially missed publications.

To comprehensively map the available evidence on manual laryngeal therapy, we included both primary intervention studies and secondary evidence syntheses, specifically meta-analyses. Data from primary intervention studies and meta-analyses were charted separately according to publication type to distinguish primary from secondary evidence and to avoid double-counting of primary studies.

### 2.2. Study Selection

All identified records were imported into the open-access tool Rayyan. Duplicates were removed, and the titles and abstracts of the remaining records were independently screened for eligibility by three researchers (Z.T., P.-M.D. and M.M.I.) according to the predefined inclusion and exclusion criteria, ensuring each article would be screened by a minimum of two reviewers. We used the AI screening and highlighting function of Rayyan, but we still screened each record manually. Any disagreements were resolved by consensus through discussion with a fourth researcher (B.B.v.L.). Full-text screening was completed independently by two researchers (Z.T., B.B.v.L.), with disagreements being resolved by consensus.

Eligible studies included human participants of any age who either had a diagnosed voice, laryngeal, swallowing, or related upper aerodigestive tract disorder, or exhibited a verifiable risk factor or functional characteristic associated with an increased likelihood of voice, laryngeal, swallowing, or related difficulties, regardless of whether a clinical diagnosis was present, and who received manual laryngeal therapy. The intervention of interest contained a range of hands-on approaches directed at the laryngeal and paralaryngeal region, including manual circumlaryngeal therapy, laryngeal manual therapy, circumlaryngeal massage, laryngeal massage, and manual therapy targeting laryngeal and paralaryngeal musculature. Studies describing other forms of direct manual manipulation of laryngeal structures, such as laryngeal shaking or laryngeal compression, were also considered eligible.

Second, to be included, studies had to report information relevant to at least one of the objectives of the review, including characteristics of the population receiving manual laryngeal therapy, the clinical indications for its use or proposed mechanisms of action, and/or outcome measures used to evaluate treatment effects. Thirdly, primary research literature, including randomized controlled trials, intervention studies, cohort studies, case–control studies, case series or studies, and mixed-methods studies, was eligible for inclusion. In addition, evidence syntheses based on meta-analyses were included separately to support evidence mapping and citation searching. Only journal publications were considered.

Studies were excluded if, first, they involved cadavers. Second, studies evaluating interventions that did not constitute a manual laryngeal intervention were also excluded. Third, studies investigating combined or eclectic therapeutic approaches in which the specific effects of manual laryngeal therapy could not be distinguished were not considered eligible. Finally, studies providing insufficient information about the population receiving manual laryngeal therapy were also excluded.

To minimise duplication of evidence, empirical intervention studies that had already been incorporated into an included meta-analysis were also excluded and considered separately from the empirical evidence synthesis in the tables and narrative. This approach was intended to avoid duplicate reporting while retaining meta-analyses as a source for evidence mapping and citation searching. The application of these criteria supported a comprehensive mapping of the available literature while maintaining a clear focus on manual interventions directed at the laryngeal region.

### 2.3. Data Extraction

The following information from the included studies were extracted: study characteristics (study title, first author, publication year, outcome measure category, and study design), population characteristics (age, laryngeal status, gender, and total number), intervention (manual laryngeal method or exercises), and effect estimates (mean, SD, *p*-values, *d*). If data were incomplete in the literature, attempts were made to contact the corresponding author(s) to obtain additional information.

### 2.4. Data Analysis, Synthesis, and Reporting

In accordance with the objectives of this scoping review, no formal methodological quality assessment or risk-of-bias assessment of the included studies was performed. The aim was to map the available evidence, characterize the existing literature, and identify research gaps rather than to evaluate the methodological rigor or determine the certainty of the evidence regarding treatment effectiveness. Nevertheless, to further characterize the nature and strength of the available evidence, the included studies were classified according to the Oxford Centre for Evidence-Based Medicine (OCEBM) Levels of Evidence [20]. This classification was used descriptively to indicate the level of evidence provided by each study and was not intended as a substitute for a formal critical appraisal of methodological quality. The review was conducted and reported in accordance with the PRISMA Extension for Scoping Reviews (PRISMA-ScR) guidelines [21].

The extracted data relating to the research questions were mapped and summarized. A qualitative analysis was used to analyze data associated with the research question separating between primary and secondary literature.

## 3. Results

### 3.1. Study Characteristics

**Figure 1:**
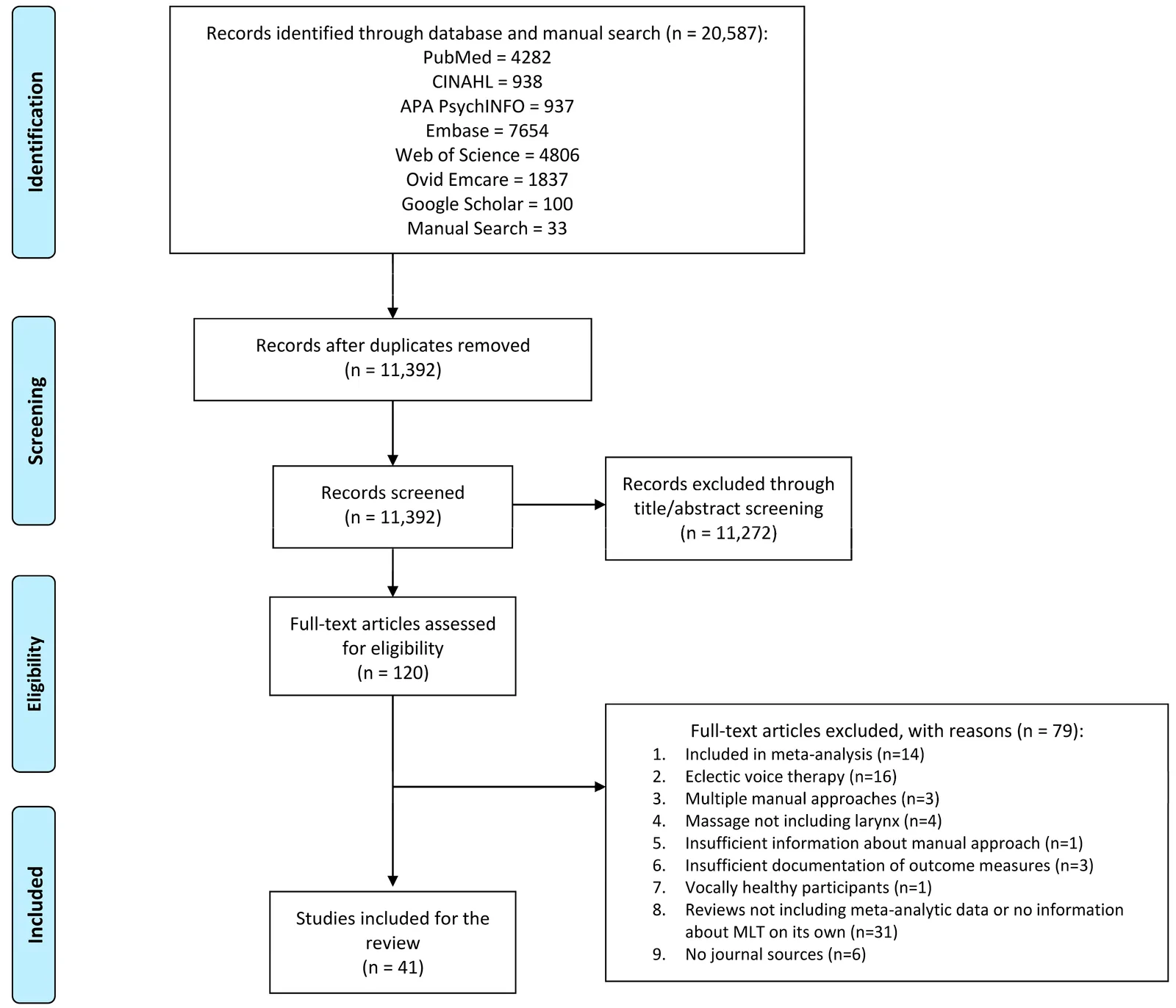
PRISMA flow diagram.

Figure 1 presents the process used to identify and select studies for this review. The initial search yielded 20,587 records. After removing duplicate entries, 11,392 studies remained for screening, of which 11,272 articles were excluded. The full text of 120 articles was retrieved for full-text screening (see Figure 1). During full-text screening, 79 articles were excluded for a variety of reasons (see Table S3), including articles that were included in a previous meta-analysis (*n* = 14), articles employing eclectic voice therapy (*n* = 16) or multiple manual approaches (*n* = 3) making it impossible to determine the effect of any single manual therapy approach, articles describing massage approaches that did not include the larynx (*n* = 4) or that did not describe the manual approach (*n* = 1) or outcome measures (*n* = 3) in sufficient detail, and one article that included a healthy population (*n* = 1). Finally, articles were excluded if they were review studies not including meta-analytic data or not including data on manual laryngeal therapy on its own within the meta-analysis (*n* = 31) or were not published in a scientific journal (i.e., theses or conference abstracts) (*n* = 6).

A total of 41 articles were included in the scoping review, 4 of which were meta-analyses and 37 of which were primary literature studies not previously included in a meta-analysis. The study details of these selected papers can be obtained in Table 1 for primary literature [22,23,24,25,26,27,28,29,30,31,32,33,34,35,36,37,38,39,40,41,42,43,44,45,46,47,48,49,50,51,52,53,54,55,56,57,58] and Table 2 for secondary literature [59,60,61,62].

The included studies represented a wide range of OCEBM evidence levels with 4 studies (10%) being level 1 (meta-analysis), 12 studies (29%) being level 2 (randomized controlled trial or observational study with dramatic effect), 5 studies (12%) being level 3 (non-randomized controlled cohort studies), 12 studies (29%) being level 4 (case-series), and 8 studies (20%) being level 5 (single case study).

### 3.2. Included Populations

Across the primary literature, a total of 1019 participants were included in this scoping review. The treatment background of all participants was voice disorders or voice complaints. Table 1 further summarizes the sample size, population characteristics, and therapy plan of each study included in the primary literature [22,23,24,25,26,27,28,29,30,31,32,33,34,35,36,37,38,39,40,41,42,43,44,45,46,47,48,49,50,51,52,53,54,55,56,57,58]. The overall gender and age distributions could not be determined because some studies did not report the gender [28,42] or age [54,56] characteristics of their participants. Furthermore, an overall summary of session intensity across the different manual laryngeal therapy approaches was not possible due to missing information in several studies [29,34,35,38,40,42,43,48,52,56]. The total number of participants included across the meta-analyses was not reported, and the corresponding participant numbers can be found in the individual references to the meta-analytic studies [59,60,61,62].

The primary literature studies included a variety of diagnoses (see details in Table 1). Primary functional voice disorders were the most common pathology (43%) [23,25,26,27,30,31,33,35,36,41,42,45,49,51,52,55], followed by singers with primary functional voice disorders (11%) [29,32,40,50], secondary functional voice disorders (8%) [23,24,25] and primary school teachers experiencing vocal fatigue (8%) [46,47,48], and mutational falsetto (5%) [38,39]. Other groups/disorders/complaints were included in single studies (30%): singers who had difficulties executing Iranian vibrato [22], ventricular dysphonia after triple coronary by-pass surgery with complications [34], vocally healthy music students with a high vocal load [37], theatre artists with vocal fatigue [28], vocal fold polyp [43], muscle tension dysphonia after coronavirus [44], complete tetraplegia [53], amateur choir singers who were vocally healthy [54], partial laryngectomy [56], elite musical performers with LPR [57], and heterogenous voice disorders [58]. Given the low prevalence of disorders other than functional voice disorders, no narrative comparison between the included pathologies could be performed. The above-describe wide variety of included populations was in contrast with the meta-analyses, which only included participants with primary and secondary functional voice disorders (Table 2).

### 3.3. Included Therapy Approaches

Similar to the variety of included populations, a variety of therapy approaches was included in the primary literature. The different therapy approaches, therapy plan and intensity are listed in Table 1. Eleven studies (29.8%) considered Laryngeal Manual Therapy (LMT), ranging in therapy intensity from one session to multiple weeks of therapy sessions. Ten studies (27.0%) discussed Manual Circumlaryngeal Therapy (MCT). Some studies considered the effect of MCT after a single treatment session (45 min to 2 h), while other studies employed the technique for multiple therapy sessions up to 5 weeks. The other laryngeal manual therapy approaches were less common. Only 3 studies (8.1%) considered Voice Massage and Cricothyroid Visor Maneuver (CVM) (8.1%), 2 studies (5.4%) considered Larynx Manipulation, Vanderbilt Manual Intervention (5.4%). Finally, a total of 6 studies (16.2%) employed other laryngeal manual therapy approaches, such as for example laryngeal shaking, compression techniques or more general approaches of massage.

The meta-analysis studies also reflected the above-listed approaches, with one meta-analysis focusing on LMT, two on MCT, and one on a combination of LMT, CVM, and MCT, showing that most of the included studies focused on MCT (see Table 2).

### 3.4. Outcome Measures

The included outcome measures across the empirical and literature studies included in this review reflect the diversity of voice assessments used in clinic and research (see Table 1 and Table 2). Some of the most commonly included measures were acoustic measures (e.g., fundamental frequency and its standard deviation, intensity, jitter, shimmer, signal-to-noise ratio, noise-to-harmonics ratio, smoothed cepstral peak prominence, Dysphonia Severity Index, Cepstral Spectral Index of Dysphonia, Acoustic Voice Quality Index, Acoustic Breathiness Index), auditory-perceptual assessment of voice (i.e., equal-appearing interval scales, visual analog scales), aerodynamic measures (maximum phonation time, *s*/*z* ratio), self-perception of the patient regarding vocal symptoms (e.g., Voice Handicap Index, Singing-Voice Handicap Index, Voice-Related Quality of Life, Vocal Tract Discomfort Scale, Patient-Specific Functional Scale), measures of musculoskeletal tension or pain (visual analog scales and equal-appearing interval scales asking patient, palpation, electromyography), and laryngeal imaging. More rarely, measures as diadochokinetic rate or range of motion of facial musculature were included as well.

Table 3 summarizes the effects of the different manual laryngeal therapy approaches in individuals with voice disorders or voice complaints. Overall, the findings indicate predominantly positive treatment effects across the investigated approaches, although the proportion of significant effects varied considerably between interventions and outcome domains.

Among the outcome parameters assessed for LMT, 46% represented primary measures without further subtests or related measurement components. Statistical significance was not applicable to 14 of all 140 outcome parameters (10%). Among the remaining 126 outcome parameters, significant positive treatment effects were observed in 67 parameters (53%). These effects were distributed across self-evaluation (39%), palpation (28%), laryngeal imaging (16%), perceptual evaluation (9%), acoustic measures (6%), and aerodynamic measures (2%). Conversely, significant negative treatment effects were identified in 6 of the 126 outcome parameters (5%), pertaining to laryngeal imaging and acoustic measures.

Among the outcome parameters assessed for MCT, 77% represented primary measures without further subtests or related measurement components. Statistical significance was not applicable to 23 of the 57 outcome parameters (40%). Among the remaining 34 outcome parameters, significant positive treatment effects were observed in 21 parameters (62%). These effects were distributed across perceptual evaluation (38%), acoustic measures (34%), self-evaluation (14%), and electromyography (14%). No significant negative treatment effects were reported.

Among the outcome parameters assessed for CVM, 81% represented primary measures without further subtests or related measurement components. Statistical significance was not applicable to 26 of the 37 outcome parameters (70%). Among the remaining 11 outcome parameters, 5 parameters (45%) demonstrated significant positive treatment effects, distributed across self-evaluation (60%), acoustic measures (20%), and aerodynamic measures (20%). No significant negative treatment effects were identified.

Among the outcome parameters assessed for Voice Massage, 31% represented primary measures without further subtests or related measurement components. Statistical significance was applicable to all 13 outcome parameters. Significant positive treatment effects were observed in 4 of the 13 outcome parameters (31%), relating to self-evaluation (50%), acoustic measures (25%), and perceptual evaluation (25%). In contrast, significant negative treatment effects were identified in 3 outcome parameters (23%), all of which concerned self-evaluation measures.

For Larynx Manipulation, statistical significance was not applicable to 1 of the 3 outcome parameters (33.3%). Of the two remaining parameters, one (50%) demonstrated a significant positive treatment effect, pertaining to aerodynamic measures. No significant negative treatment effects were reported.

Finally, for the Vanderbilt Manual Intervention, statistical significance was not applicable to 13 of the 15 outcome parameters (87%). Neither of the two remaining outcome parameters demonstrated a significant positive treatment effect, and no significant negative treatment effects were identified.

The meta-analyses primarily focused on outcome measures that were most frequently assessed, including perceptual evaluation (overall severity), acoustic measures of voice quality, vocal function, and articulation, as well as self-evaluation measures (pain and intensity of laryngeal symptoms) (see Table 4). All meta-analyses investigating MCT demonstrated significant treatment effects across the assessed perceptual and acoustic measures of voice quality. Furthermore, significant positive treatment effects were observed for selected measures of vocal function (i.e., I-low) and articulation (i.e., F1, F2, and F2 slope) following MCT. In contrast, the meta-analyses investigating LMT revealed no significant changes in perceived voice quality or self-evaluation measures compared with voice exercises or transcutaneous electrical nerve stimulation.

## 4. Discussion

The goal of this scoping review was to map the literature on manual laryngeal therapy, focusing on the populations and clinical indicators addressed, as well as the outcome measures used to assess treatment outcomes and their associated effect sizes. Across 41 studies (i.e., 4 meta-analyses and 37 primary literature studies), we found that manual laryngeal therapy is mostly used in patient populations with primary and secondary functional disorders, though it also has shown benefit in other populations, including healthy populations at risk for voice disorders. Dysphagia or swallowing symptoms were not tested in isolation with manual laryngeal therapy approaches (see Table S3) and were thus not part of the present scoping review. Due to the low prevalence of the other voice disorders, no further conclusions on the clinical indications for manual laryngeal therapy could be drawn. Across the included studies, MCT and LMT were the most evaluated treatment approaches. Overall, a positive effect of manual laryngeal therapies was observed across a variety of outcome measures. The highest proportion of significant positive treatment effects was observed for MCT (62%), followed by LMT (53%), Larynx Manipulation (50%), CVM (45%), and Voice Massage (31%). No significant positive treatment effects were observed for Vanderbilt Manual Intervention (0%). However, the result for the Larynx Manipulation was based on only one significant positive effect among two evaluated outcome parameters and therefore has limited weight in the overall assessment of the current review. Significant negative treatment effects were observed for Voice Massage (23%) and LMT (5%).

This scoping review showed that manual laryngeal therapy is mostly used in primary and secondary functional disorders, as over 50% of the included empirical studies and all of the meta-analyses investigated these patient populations. Functional voice disorders are some of the most commonly reported voice disorders [63]. A primary functional voice disorder is characterized by, for example, vocal symptoms as voice quality changes, pain or various symptoms of discomfort in the (para)laryngeal region, or reduced pitch range, in the absence of an identifiable organic pathology, with hyperfunctional muscular tension often considered as a hallmark contributing factor [64,65]. On the other hand, secondary functional voice disorders present with similar symptoms and tension, but are accompanied by underlying structural abnormities [65]. While the exact working mechanism of manual laryngeal therapy remains unclear, it is often touted to directly address muscular hyperfunction [5]. Given the prominence of hyperfunctional muscle activity in both primary and secondary functional voice disorders, it is not surprising that these populations were the focus of a substantial proportion of the studies included in this review.

For the treatment of voice disorders caused by, or associated with, hyperfunctional muscle tension, the findings of the present review suggest that passive manual laryngeal therapy performed at rest position, without targeted voice exercises, may be less effective. This finding is consistent with those from a network meta-analysis comparing 24 voice treatment approaches and evaluating their effects on perceived dysphonia [62]. MCT provides an example of a treatment approach combining manual techniques with voice exercises. Across the included primary studies [33,34,35,36,37,38,39,40,41,42] and meta-analyses [60,61,62], MCT was associated with favorable outcomes in acoustically and auditory-perceptually assessed dysphonia. This contrasts with approaches such as LMT, which involve manual treatment while the patient is at rest [11]. Another meta-analytic evaluation found no evidence that LMT was superior to standard voice exercises or transcutaneous electrical nerve stimulation [59].

In its original form, MCT combines passive manual treatment with two principal manual techniques, namely hyoid push-back and thyroid pull-down. These two techniques incorporate voice exercises, typically humming at a relatively low pitch and high loudness [8,12]. CVM represents an advancement of MCT, including a manual technique focused on the cricothyroid visor mechanism during high phonation, such as humming and vowel production [66]. This technique is based on a sub-concept of Lieberman’s Larynx Manipulation approach [9,10]. However, the extent to which this addition improves treatment outcomes remains unclear. The available evidence on CVM is limited to case reports and pilot studies. The Vanderbilt Manual Intervention also combines manual elements of MCT and Larynx Manipulation, but uses manual techniques with the patient at rest and does not include voice exercises [51]. As with CVM, the findings of the present review of the Vanderbilt Manual Intervention did not allow a clear conclusion regarding the efficacy of this manual laryngeal approach, given that only limited effects could be evaluated for the Voice Handicap Index. In one larger study, standard voice therapy incorporating voice exercises was the only approach producing significant results as the on this measure, rather than the manual intervention.

Nevertheless, LMT may be useful for addressing laryngeal discomfort and other symptoms in voice disorders. Findings from at least two studies included improvements in self-evaluated outcomes including reduced musculoskeletal pain in the temporal region, larynx, back of the neck, upper back, and lower back, as well as changes in the Singing Voice Handicap Index). Improvements were also reported in perceptual outcomes (i.e., overall voice quality and strain), palpation findings (i.e., reduced tension in the sternocleidomastoid muscle and changes in lateral movement, the submental and infrahyoid areas, and the cricothyroid area), laryngeal imaging findings (i.e., improvement in the muscle tension dysphonia pattern), and acoustic measure (i.e., reduced fundamental frequency in male participants).

Taken together, the findings of the present review suggest that combining MCT and LMT may be a useful approach within manual laryngeal therapy for patients with voice disorders. Future development of manual laryngeal therapy concepts could involve tailoring specific manual techniques to diagnostic findings and outcome measures associated with increased muscle tension. Potential targets include breathiness or aphonia (e.g., thyroid pull-down), roughness (e.g., hyoid push-back), high pitch (e.g., thyroid pull-down or laryngeal depression), vocal fatigue or tiredness, and other localized laryngeal discomfort (e.g., massage of the sternocleidomastoid muscle, supralaryngeal area, or circumlaryngeal hotspot areas). Pain could also be addressed using circumlaryngeal massage of identified hotspot areas. These symptoms and findings could be assessed through palpation [17,19,67], acoustic measures [67], standardized voice-symptom questionnaires [67], or other self-report rating systems. In addition to established approaches such as MCT and LMT, a new treatment concept based on diagnosis-specific outcome measures, as described above, could investigate a broader repertoire of targeted manual laryngeal techniques and should categorize the various techniques to increase reliability and reproducibility. Their efficacy and effectiveness would need to be evaluated systematically. Finally, the amount of force applied during manual techniques also warrants further investigation. Clinicians use different handling procedures and apply varying levels of pressure, which may influence treatment effects but have not been adequately investigated in manual laryngeal therapy. MCT and Larynx Manipulation have often involved relatively high force to reduce tension in the neck and surrounding areas, as described in practical workshops by Lieberman and Roy. This contrasts with the Vocal Manual Therapy Intervention [57], which follows a low-force manual therapy approach intended to achieve a substantial therapeutic effect [68].

This scoping review presented with some limitations. The current review only included studies if the study reported on the outcome of a single manual laryngeal therapy approach. This enabled us to consider each manual laryngeal therapy approach specifically. However, this also means that studies investigating eclectic voice therapy approaches were excluded. Consequently, some populations for whom manual laryngeal therapy may be beneficial might not have been captured in this review. In the treatment of dysphonia, eclectic approaches combining manual laryngeal therapy concepts with symptomatic treatment approaches, such as facilitating techniques, and additional components, including vocal hygiene and electrostimulation (where applicable), are common and appear to yield greater effects than other treatment approaches or manual circumlaryngeal therapy alone [62]. Moreover, while we did include grey literature in the scoping review via the inclusion of the first 100 results on Google Scholar, our search results could possibly have missed non-traditionally published sources, leading to publication bias.

While the scoping nature of this review allows us to provide a broad overview of the treatment populations and treatment outcomes of manual laryngeal therapy, the conclusions regarding treatment effectiveness need to be considered with some caveats. As is typical in a scoping review [20], we did not assess the quality of the included articles. Though the tables provide general information on treatment population, manual laryngeal therapy approaches, and their effect on outcome measures, the findings should only be interpreted in a descriptive manner without conclusions on the strength of evidence supporting the different manual therapy approaches. Moreover, the substantial variability in methodological approaches, ranging from case studies to randomized controlled trials, along with the diverse array of reported outcome measures, limited the direct comparison of the included studies. Additionally, several studies either lacked statistical analyses or provided insufficient methodological detail, complicating the interpretation and reporting of studies for this scoping review. While these limitations constrain the conclusions for the review, they also highlight the need for greater standardization in voice assessment and outcome protocols and more transparent reporting of research methodologies and outcomes.

Future development of manual laryngeal therapy should consider diagnosis- and symptom-specific selection of manual techniques and standardized outcome measures, with systematic categorization of techniques to improve treatment reproducibility. Further research is also needed to determine the contribution of individual manual techniques, the role of treatment force, and the effectiveness of combined manual and behavioral approaches. Well-designed controlled studies using standardized diagnostic and outcome measures will be essential to establish the efficacy and clinical effectiveness of these approaches and to support their integration into evidence-based voice therapy.

## 5. Conclusions

This scoping review mapped the current evidence on manual laryngeal therapy across 41 studies and identified a predominantly positive pattern of treatment effects across a range of clinical and patient-reported outcomes. The available evidence is concentrated in populations with primary and secondary functional voice disorders, with MCT and LMT representing the most frequently investigated approaches. Overall, the findings suggest that manual laryngeal therapy may be particularly relevant when manual techniques are integrated with active voice exercises, as exemplified by MCT, whereas passive manual treatment alone may have more limited effects on dysphonia. LMT may nevertheless provide benefits for laryngeal and musculoskeletal discomfort and other associated symptoms.

## Figures and Tables

**Table 1:** Characteristics of empirical trials in the scoping review, ranging from most to least evaluated manual laryngeal approaches.

Study	OCEBM Level of Evidence [20]	Sample Size	Age (in Years)/Gender of the Treatment Groups	Population	Assessment	Therapy Plan	Session Intensity
**Laryngeal Manual Therapy (*n* = 11)**							
Salehi & Barkmeier-Kraemer [22]	4	4	Mean/range 35.25 (28–40) F/M = 1/3	Singers who had difficulties to execute Iranian vibrato (tahrir)	Acoustic	20 therapy sessions for 6 to 7 weeks of LMT	15 min
Reimann et al. [23]	3	30	Mean/range 25.08 (18–45) F/M = 24/6	Primary and secondary functional voice disorders; vocally healthy	Perceptual Evaluation; Self-evaluation; Acoustic	A single treatment approach of LMT or control group without therapy for vocally healthy	20 min
Siqueira et al. [24]	2	20	Mean/range 38.33 (22–54) F/M = 20/0	Secondary functional voice disorders	Acoustic	12 therapy sessions of LMT, or TENS	20 min
Conde et al. [25]	2	30	Mean/range 28.7 (18–45) F/M = 30/0	Primary and secondary functional voice disorders	Self-evaluation; Perceptual Evaluation; Acoustic	A single treatment approach of LMT or TENS	20 min
Das et al. [26]	4	10	Mean/range 28.7 (20–40) F/M = 10/0	Primary functional voice disorder	Palpation; Perceptual Evaluation; Acoustic	A single treatment approach of LMT	45 min
Fallah et al. [27]	4	12	Mean/range 38.33 (22–54) F/M = 12/0	Primary functional voice disorder	Perceptual Evaluation; Laryngeal Imaging; Self-Evaluation	5 therapy sessions for 2.5 weeks of LMT	25 min
Jain & Ravu [28]	4	42	Mean/range 23 (18–25) F/M = N/A	Theatre artists with vocal fatigue	Palpation	A single treatment approach of LMT	5 to 10 min
Ahmadi et al. [29]	4	32	Mean/range 36.75 (20–60) F/M = 8/24	Singers who had primary functional voice disorder	Perceptual Evaluation; Laryngeal Imaging; Self-Evaluation; Acoustic	10 therapy sessions for 3 months of LMT	N/A
Hashemnia et al. [30]	4	4	Mean/range 36.75 (21–25) F/M = 4/0	Primary functional voice disorder	Acoustic; Aerodynamic	12 therapy sessions for 2 weeks of LMT	20 min
Lemos et al. [31]	2	28	Mean/range 41.47 (18–55) F/M = 28/0	Primary functional voice disorder	Perceptual Evaluation; Aerodynamic; Self-evaluation; Palpation	12 therapy sessions for 2 weeks of LMT or TENS or combined therapy	24 min
Ahmadi et al. [32]	2	31	Mean/range 34.48 (21–63) F/M = 0/31	Singers with primary functional voice disorder	Acoustic; Laryngeal Imaging; Perceptual Evaluation; Self-evaluation	8 therapy sessions for 4 weeks of LMT or LMT + ET	1 h
**Manual Circumlaryngeal Therapy (*n* = 10)**							
Roy & Tasko [33]	4	8	Mean/range 39 (20–58) F/M = 8/0	Primary functional voice disorder	Acoustic; Perceptual Evaluation	A single treatment approach of MCT	45 min to 2 h
Roy [34]	5	1	61 F/M = 0/1	Ventricular dysphonia after triple coronary-bypass surgery with complications	Acoustic; Perceptual Evaluation	8 therapy sessions for 8 weeks	N/A
Roy & Hendarto [35]	4	40	Mean/range 47.98 (26–79) F/M = 40/0	Primary functional voice disorder	Acoustic; Perceptual Evaluation	A single treatment approach of MCT	N/A
Russel [36]	5	1	6 F/M = 1/0	Primary functional voice disorder	Acoustic; Perceptual Evaluation	A single treatment approach of MCT	2 h
D’haeseleer et al. [37]	2	16	Mean/range 21.4 (19–25) F/M = 11/5	Vocally healthy music students with high vocal load	Acoustic	A single treatment approach of MCT or VR	20 min
Roy et al. [38]	4	12	Mean/range 17.5 (14–25) F/M = 0/12	Mutational Falsetto	Acoustic; Perceptual Evaluation	A single treatment approach of MCT	N/A
Barsties v. Latoszek [39]	5	1	18 F/M = 0/1	Mutational Falsetto	Acoustic; Self-evaluation	2 therapy sessions for 2 weeks of MCT	45 min
Pani et al. [40]	5	1	18 F/M = 1/0	Singer with primary functional voice disorder	Acoustic; Perceptual evaluation; Laryngeal Imaging; Aerodynamic; Palpation	A single treatment approach of MCT	N/A
Shabani et al. [41]	2	10	Mean/range 33.8 (24–44) F/M = 0/10	Primary functional voice disorder	Acoustic; surface EMG	10 therapy sessions for 5 weeks of MCT or MCT + TENS	40 min
Behroozi et al. [42]	2	40	Mean/SD 38.6/4.6 F/M = N/A	Primary functional voice disorder	Acoustic; Self-evaluation	10 therapy sessions for 5 weeks of MCT or combined therapy with medical herbs (Echinops) 2× per day for 4 weeks	N/A
**Cricothyroid Visor Maneuver (*n* = 3)**							
Dehqan [43]	5	1	48 F/M = 1/0	Vocal fold polyp	Perceptual Evaluation; Laryngeal Imaging; Self-Evaluation; Acoustic	10 therapy sessions for 3.5 weeks of CVM	N/A
Moein et al. [44]	5	1	55 F/M = 0/1	Post-COVID-19 and primary functional voice disorder	Acoustic; Perceptual Evaluation; Aerodynamic; Laryngeal Imaging; Self-Evaluation	10 therapy sessions for 3.5 weeks of CVM	30 min
Nasrin et al. [45]	4	6	Mean/range 38.5 (30–45) F/M = 4/2	Primary functional voice disorder	Acoustic; Self-evaluation; Perceptual Evaluation	5 therapy sessions for 2.5 weeks of CVM	30 min
**Voice Massage (*n* = 3)**							
Laukkanen et al. [46]	2	90	Mean/SD 41.1/8.5 F/M = 90/0	Primary school teachers who showed regular vocal fatigue	Self-evaluation	5 therapy sessions for 5 weeks of Voice Massage or Voice training, or a single treatment of 3 h VH	1 h
Leppänen et al. [47]	2	59	Mean/range 40.6 (26–57) F/M = 56/0	Primary school teachers who showed regular vocal fatigue	Acoustic; Perceptual Evaluation; Self-evaluation	5 therapy sessions for 5 weeks of Voice Massage and/or a single treatment of 3 h VH	1 h
Leppänen et al. [48]	2	49	Mean/SD 41.1/8.5 F/M = 49/0	Primary school teachers who showed regular vocal fatigue	Self-evaluation	Follow-up assessment after 1 year from Laukkanen et al. [46]	N/A
**Larynx Manipulation (*n* = 2)**							
Van Rijsen [49]	5	1	55 F/M = 0/1	Primary functional voice disorder	Palpation; Self-evaluation	6 therapy sessions for 6 weeks of Larynx Manipulation	30 min
Ahmadi et al. [50]	2	51	Mean/range 37.6 (20–65) F/M = 11/40	Singers who had primary functional dysphonia	Aerodynamic; Self-evaluation; Laryngeal imaging	13 therapy sessions for 13 weeks of Larynx Manipulation, or breathing exercises, or combined therapy, or a control group without therapy	25 min to 50 min
**Vanderbilt Manual Intervention (*n* = 2)**							
Tomlinson & Archer [51]	4	9	Mean/range 52 (40–88) F/M = 9/0	Primary functional voice disorder	Self-evaluation; Functional status; Range of Motion	11–18 therapy sessions for 9 weeks of VMI	60 min
Craig et al. [52]	3	153	Mean/range 47.88 (30–59) F/M = 104/49	Primary functional voice disorder	Self-evaluation	8 therapy sessions for 39 to 114 days of VMI or at least 2 therapy sessions for 43 to 119 days for voice therapy or combined therapy (61 to 140 days)	N/A
**Others (*n* = 1)**							
Sullivan et al. [53]	5	1	25 F/M = 1/0	Complete Tetraplegia	Acoustic; Self-evaluation	20 therapy sessions for 4 weeks of traditional Chinese massage of laryngeal region and surrounding neck, face, and shoulder musculature	15 min
Ternström et al. [54]	2	31	Mean/range N/A F/M = 22/9	Amateur choir singers who were vocally healthy	Acoustic	A singe approach of body massage or lying on a bed	30 min
Voerman et al. [55]	3	116	Mean/range 42 (10–80) F/M = 107/9	Primary functional voice disorder	Self-evaluation	1–3 therapy sessions of Laryngeal Shaking, or Mental Imagery, or combined therapy	10 min to 2 h
Mumović [56]	3	66	Mean/range N/A F/M = 0/66	Partial laryngectomy	Perceptual Evaluation; Acoustic	A single approach of laryngeal compression or standard voice therapy for at least 6 weeks with 2 to 3 therapy sessions a week	N/A
Flock & King [57]	4	2	Mean/range 26 (23–29) F/M = 1/1	Elite musical theatre performers who had LPR	Self-evaluation	4 therapy sessions for 4 weeks of VMTI	11 min
de Brito et al. [58]	3	21	Mean/range 38.76 (24–56) F/M = 21/0	Heterogenous voice disorders	Acoustic; Perceptual Evaluation; Self-evaluation; Palpation	A single approach of three techniques by the American Osteopathic Association and VR	10 min

*Abbreviation*: CVM = Cricothyroid Visor Maneuver; EMG = Electromyography; ET = Edge Therapy; LMT = Laryngeal Manual Therapy; LPR = Laryngopharyngeal Reflux; MCT = Manual Circumlaryngeal Therapy; SD = Standard Deviation; TENS = Transcutaneous Electrical Nerve Stimulation; VH = Vocal Hygiene; VR = Voice Rest; VMI = Vanderbilt Manual Intervention; VMTI = Vocal Manual Therapy Intervention.

**Table 2:** Characteristics of meta-analyses studies in the scoping review.

Study	Type of Meta-Analysis (Level 1 by OCEBM [20])	N of Studies	Population	Assessment
Ribeiro et al. [59]	Standard (pairwise) meta-analysis	2	Primary and secondary functional voice disorders	Perceptual Evaluation; self-evaluation
Kim [60]	Single treatment meta-analysis	10	Primary functional voice disorder	Acoustic
Barsties v. Latoszek et al. [61]	Single treatment meta-analysis	6	Primary functional voice disorder	Acoustic
Barsties v. Latoszek et al. [62]	Network meta-analysis	30	Primary and secondary functional voice disorders	Perceptual Evaluation

**Table 3:** Results from empirical studies. Ranging from most to lowest evaluated manual laryngeal approaches.

Study	Group of Comparison	Outcome Measure	Effect Estimation	*p*-Value
**Laryngeal Manual Therapy (*n* = 11)**				
Salehi & Barkmeier-Kraemer [22]	Laryngeal Manual Therapy	Vibrato rate F0 extent SPL extent	MD: 3 Hz MD: −1.85% MD: 8.7%	N/A
Reimann et al. [23]	Laryngeal Manual Therapy (*n* = 15), vocally healthy control group (*n* = 15)	MPI: temporal region (100 mm VAS) MPI: masseter (100 mm VAS) MPI: suprahyoid (100 mm VAS) MPI: larynx (100 mm VAS) MPI: front of the neck (100 mm VAS) MPI: back of the neck (100 mm VAS) MPI: shoulders (100 mm VAS) MPI: upper back (100 mm VAS) MPI: elbows (100 mm VAS) MPI: fists/hands/fingers (100 mm VAS) MPI: lower back (100 mm VAS) MPI: hip/thighs (100 mm VAS) MPI: knees (100 mm VAS) MPI: ankles/feet (100 mm VAS)	LMT = MD: −7.0 mm CG = MD: 2.6 mm LMT = MD: −6.7 mm CG = MD: −3.6 mm LMT = MD: −3.7 mm CG = MD: 4.8 mm LMT = MD: −15.0 mm CG = MD: 0.0 mm LMT = MD: −6.0 mm CG = MD: 0.0 mm LMT = MD: −10.9 mm CG = MD: −3.3 mm LMT = MD: −5.6 mm CG = MD: −4.9 mm LMT = MD: −9.9 mm CG = MD: −1.1 mm LMT = MD: −2.4 mm CG = MD: 0.3 mm LMT = MD: −7.0 mm CG = MD: 0.7 mm LMT = MD: −8.5 mm CG = MD: −4.8 mm LMT = MD: −3.1 mm CG = MD: 0.1 mm LMT = MD: −5.7 mm CG = MD: −0.2 mm LMT = MD: −0.7 mm CG = MD: −3.5 mm	**0.028** 0.593 0.069 0.590 0.294 1.00 **0.026** 1.00 0.260 1.00 **0.005** 1.00 0.086 0.138 **0.016** 0.593 0.144 1.00 **0.036** 1.00 **0.008** 0.068 **0.028** 1.00 0.161 1.00 0.345 0.285
		*Results are shown only for vowel/a/* Overall perceived voice quality (pairwise rating to baseline)	LMT = improved: 53% LMT = worsened: 40% LMT = unchanged: 7% CG = improved: 20% CG = worsened: 20% CG = unchanged: 60%	0.791 1.00
		Perceived roughness (pairwise rating to baseline)	LMT = improved: 14% LMT = worsened: 33% LMT = unchanged: 53% CG = improved: 7% CG = worsened: 7% CG = unchanged: 86%	0.688 1.00
		Perceived breathiness (pairwise rating to baseline)	LMT = improved: 27% LMT = worsened: 33% LMT = unchanged: 40% CG = improved: 7% CG = worsened: 20% CG = unchanged: 73%	0.727 0.625
		Perceived strain (pairwise rating to baseline)	LMT = improved: 33.3% LMT = worsened: 33.3% LMT = unchanged: 33.3% CG = improved: 7% CG = worsened: 7% CG = unchanged: 86%	1.00 1.00
		Perceived instability (pairwise rating to baseline)	LMT = improved: 27% LMT = worsened: 27% LMT = unchanged: 46% CG = improved: 27% CG = worsened: 20% CG = unchanged: 53%	1.00 1.00
		Jitter on vowel/a/	LMT = MD: 0.07% CG = MD: −0.82%	0.800 **0.033**
		Shimmer on vowel/a/	LMT = MD: 0.04% CG = MD: −0.86%	0.845 0.098
		NHT on vowel/a/	LMT = MD: 0.0 dB CG = MD: 0.01 dB	0.783 0.566
		Self-evaluated sensation after treatment: Voice	LMT = positive: 67% LMT = negative: 20% LMT = unchanged: 13% CG = positive: N/A CG = negative: N/A CG = unchanged: N/A	0.092 N/A
		Self-evaluated sensation after treatment: Larynx	LMT = positive: 67% LMT = negative: 13% LMT = unchanged: 20% CG = positive: 40% CG = negative: 20% CG = unchanged: 40%	**0.039** 0.500
		Self-evaluated sensation after treatment: Articulation	LMT = positive: 47% LMT = negative: 0% LMT = unchanged: 53% CG = positive: 13% CG = negative: 0% CG = unchanged: 87%	**0.016** 0.500
		Self-evaluated sensation after treatment: Breathing	LMT = positive: 20% LMT = negative: 13% LMT = unchanged: 67% CG = positive: 40% CG = negative: 33% CG = unchanged: 27%	0.092 1.00
Siqueira et al. [24]	Laryngeal Manual Therapy (*n* = 10), Transcutaneous Electrical Nerve Stimulation (*n* = 10)	*Results are shown only for vowel/a/* DDCavR DDCavP DDCdpP DDCcvP DDCjiitP DDCcvI	LMT = DPP: 0.19 s TENS = DPP: −15.35 s LMT = DPP: 0.09 s TENS = DPP: 0.31 s LMT = DPP: 8.22 ms TENS = DPP: −8.11 ms LMT = DPP: 3.85% TENS = DPP: −3.12% LMT = DPP: 1.25% TENS = DPP: −1.2% LMT = DPP: −0.03% TENS = DPP: −0.21%	0.743 0.203 0.938 0.225 0.580 0.478 0.617 0.572 0.471 0.398 0.768 0.859
Conde et al. [25]	Laryngeal Manual Therapy (*n* = 15), Transcutaneous Electrical Nerve Stimulation (*n* = 15)	MPI: back of the neck (100 mm VAS) MPI: shoulders (100 mm VAS) MPI: upper back (100 mm VAS) MPI: elbow (100 mm VAS) MPI: fists/hand/fingers (100 mm VAS) MPI: lower back (100 mm VAS) MPI: temporal region (100 mm VAS) MPI: masseter (100 mm VAS) MPI: submandibular area (100 mm VAS) MPI: larynx (100 mm VAS) MPI: front of the neck (100 mm VAS)	LMT = MD: −11.87 mm TENS = MD: −8.07 mm LMT = MD: −6.26 mm TENS = MD: −8.73 mm LMT = MD: −7.47 mm TENS = MD: −9.86 mm LMT = MD: −2.46 mm TENS = MD: −3.47 mm LMT = MD: −6.33 mm TENS = MD: −1.60 mm LMT = MD: −8.60 mm TENS = MD: −8.60 mm LMT = MD: −7.74 mm TENS = MD: −2.33 mm LMT = MD: −6.80 mm TENS = MD: −3.54 mm LMT = MD: −3.47 mm TENS = MD: 0.87 mm LMT = MD: −15.74 mm TENS = MD: −6.40 mm LMT = MD: −8.54 mm TENS = MD: −4.74 mm	**0.012** **0.043** **0.028** **0.042** **0.050** **0.012** 0.109 0.180 0.051 0.144 **0.012** **0.018** **0.028** 0.285 0.063 **0.027** 0.351 0.465 0.051 0.138 0.263 **0.034**
		*Results are shown only for vowel/a/* Overall perceived voice quality (pairwise rating to baseline)	LMT = improved: 60% LMT = worsened: 40% LMT = unchanged: 0% TENS = improved: 60% TENS = worsened: 20% TENS = unchanged: 20%	**0.002** 0.146
		Perceived roughness (pairwise rating to baseline)	LMT = improved: 27% LMT = worsened: 7% LMT = unchanged: 66% TENS = improved: 47% TENS = worsened: 13% TENS = unchanged: 40%	0.140 0.180
		Perceived breathiness (pairwise rating to baseline)	LMT = improved: 20% LMT = worsened: 27% LMT = unchanged: 53% TENS = improved: 40% TENS = worsened: 20% TENS = unchanged: 40%	0.143 0.580
		Perceived strain (pairwise rating to baseline)	LMT = improved: 40% LMT = worsened: 33% LMT = unchanged: 27% TENS = improved: 47% TENS = worsened: 20% TENS = unchanged: 33%	**0.001** 0.344
		Perceived instability (pairwise rating to baseline)	LMT = improved: 33% LMT = worsened: 20% LMT = unchanged: 47% TENS = improved: 40% TENS = worsened: 0% TENS = unchanged: 60%	0.796 **0.031**
		F0	LMT = MD: 2.54 Hz TENS = MD: −3.82 Hz	0.335 0.153
		Shimmer	LMT = MD: 0.21% TENS = MD: −0.16%	0.290 0.325
		Jitter	LMT = MD: 0.27% TENS = MD: −0.19%	0.349 0.569
		NHR	LMT = MD: 0.0 dB TENS = MD: 0.0 dB	0.645 0.734
		*More parameters cannot be presented because of incomplete data presentation to create MD values or pairwise differences*		
Das et al. [26]	Laryngeal Manual Therapy	VHI-T	MD: −22	**<0.05** (post-therapy vs. baseline)
		VHI-p	MD: −11	**<0.05** (post-therapy vs. baseline)
		VHI-f	MD: −8	>0.05 (post-therapy vs. baseline)
		VHI-e *More parameters cannot be presented because of lack of quantitative data presentation by Das et al.* [26]	MD: −8	>0.05 (post-therapy vs. baseline)
Fallah et al. [27]	Laryngeal Manual Therapy	Laryngeal Imaging: ML-compression	DPP: 25%	N/A
		Laryngeal Imaging: AP-compression	DPP: −13%	N/A
		Laryngeal Imaging: ML-AP-compression	DPP: −29%	N/A
		Laryngeal Imaging: no compression	DPP: 17%	N/A
		VTD-f	DPP: −2.75	0.113
		VTD-s	DPP: −1.5	0.722
		*Results are shown only for vowel/a/* Overall perceived voice quality (VAS 100 mm)	DPP: −17.5 mm	**0.007**
		Perceived strain (VAS 100 mm)	DPP: −19.8 mm	**0.004**
Jain & Ravu [28]	Laryngeal Manual Therapy	Palpation: sternocleidomastoid muscle-right (4-EAI scale)	MD: −1.1	**<0.0001**
		Palpation: sternocleidomastoid muscle-left (4-EAI scale)	MD: −0.65	**<0.0001**
		Palpation: supralaryngeal area (4-EAI scale)	MD: −0.65	**<0.0001**
		Palpation: lateral movement area (4-EAI scale)	MD: −0.8	**<0.0001**
		Palpation: laryngeal high position (4-EAI scale)	MD: 0.07	0.083
Ahmadi et al. [29]	Laryngeal Manual Therapy	Laryngeal Imaging: presence of no MTD pattern	Before: 0% After: 31%	**<0.05** (in total)
		Laryngeal Imaging: presence of MTD pattern type 1	Before: 6% After: 22%	
		Laryngeal Imaging: presence of MTD pattern type 2	Before: 9% After: 9%	
		Laryngeal Imaging: presence of MTD pattern type 3	Before: 56% After: 31%	
		Laryngeal Imaging: presence of MTD pattern type 4	Before: 16% After: 6%	
		Laryngeal Imaging: presence of MTD pattern type 5	Before: 13% After: 0%	
		F0 (male) on vowel/i/ F0 (female) on vowel/i/ Jitter on vowel/i/ Shimmer on vowel/i/ HNR on vowel/i/	MD: −14.45 Hz MD: −4.32 Hz MD: −0.02% MD: 2.72% MD: −8.52 dB	**0.009** 0.484 0.57 **0.000** **0.000**
		Perceived rating of the operatic singing voice (10-EAI scale)	MD: 1.9	**0.000**
		SVHI:	MD: −10.87	**0.002**
Hashemnia et al. [30]	Laryngeal Manual Therapy	*Results are shown only for vowel/a/* F0 Jitter Shimmer HNR CPP MPT	DPP: 10.88 Hz DPP: 0.01% DPP: −0.46% DPP: 1.11 dB DPP: 0.78 dB DPP: 0.78 s	N/A
Lemos et al. [31]	Laryngeal Manual Therapy (*n* = 10), Transcutaneous Electrical Nerve Stimulation (*n* = 9), Combined Therapy (*n* = 9)	Overall perceived voice quality (4-EAI scale)	LMT = MD: −0.42 TENS = MD: −0.46 CT = MD: −0.35	**0.011** 0.066 **0.038**
		Perceived roughness (4-EAI scale) Perceived breathiness (4-EAI scale) Perceived strain (4-EAI scale) MPT S/Z Ratio VFI VSS MPI: back of the neck (100 mm VAS) MPI: temporal region (100 mm VAS) MPI: upper back (100 mm VAS) MPI: lower back (100 mm VAS) MPI: masseter (100 mm VAS) MPI: submandibular area (100 mm VAS) MPI: larynx (100 mm VAS) MPI: front of the neck (100 mm VAS)	LMT = MD: −0.20 TENS = MD: −0.30 CT = MD: −0.49 LMT = MD: −0.35 TENS = MD: −0.77 CT = MD: 0.44 LMT = MD: −0.20 TENS = MD: −0.22 CT = MD: 0.46 LMT = MD: 2.42 s TENS = MD: 2.95 s CT = MD: 2.41 s LMT = MD: −0.07 TENS = MD:−0.13 CT = MD: −0.09 LMT = MD: −24.6 TENS = MD: −21.6 CT = MD: −20.3 LMT = MD: −35.5 TENS = MD: −31.4 CT = MD: −26.6 LMT = MD: −38.2 mm TENS = MD: −36.8 mm CT = MD: −35.6 mm LMT = MD: −56.0 mm TENS = MD: −28.2 mm CT = MD: −10.3 mm LMT = MD: −33.0 mm TENS = MD: −38.8 mm CT = MD: −38.7 mm LMT = MD: −32.1 mm TENS = MD: −12.6 mm CT = MD: −34.9 mm LMT = MD: −32.7 mm TENS = MD: −14.1 mm CT = MD: −22.0 mm LMT = MD: −12.5 mm TENS = MD: −19.8 mm CT = MD: −25.3 mm LMT = MD: −37.4 mm TENS = MD: −11.9 mm CT = MD: −39.5 mm LMT = MD: −16.4 mm TENS = MD: −28.1 mm CT = MD: −31.1 mm	0.113 0.264 0.068 0.134 **0.017** 0.051 0.134 **0.017** 0.051 **0.002** **0.01** **0.007** 0.696 0.112 0.416 **<0.001** **<0.001** **<0.001** **<0.001** **0.001** **<0.001** **0.007** **<0.001** **0.001** **0.022** 0.065 0.194 **0.005** **0.001** **0.001** **0.005** **0.026** **0.002** **0.036** **0.004** **0.044** 0.213 0.062 **0.039** **0.003** 0.428 **0.007** 0.059 **0.015** **0.030**
		Palpation: submental area at rest (4-EAI scale)	LMT = MD: −0.62 TENS = MD: −0.71 CT = MD: −0.60	**0.013** **0.024** 0.051
		Palpation: infrahyoid area-left at rest (4-EAI scale)	LMT = MD: −0.29 TENS = MD: −0.52 CT = MD: −1.03	**0.038** **0.010** **0.034**
		Palpation: infrahyoid area-right at rest (4-EAI scale)	LMT = MD: −0.49 TENS = MD: −0.40 CT = MD: −0.86	**0.026** **0.009** 0.096
		Palpation: cricothyroid–left at rest (4-EAI scale)	LMT = MD: −0.80 TENS = MD: −0.48 CT = MD: −1.42	**0.001** 0.218 **<0.001**
		Palpation: cricothyroid-right at rest (4-EAI scale)	LMT = MD: −0.91 TENS = MD: −0.79 CT = MD: −1.77	**<0.001** **0.008** **<0.001**
		Palpation: sternocleidomastoid muscle–left at rest (4-EAI scale)	LMT = MD: −1.14 TENS = MD: −0.61 CT = MD: −0.68	0.058 **<0.001** **0.003**
		Palpation: sternocleidomastoid muscle–right at rest (4-EAI scale)	LMT = MD: −1.00 TENS = MD: −0.40 CT = MD: −0.77	0.081 **0.040** **0.016**
		Palpation: submental area during voicing (4-EAI scale)	LMT = MD: −0.91 TENS = MD: −0.69 CT = MD: −0.94	**<0.001** **0.022** **0.002**
		Palpation: infrahyoid area-left during voicing (4-EAI scale)	LMT = MD: −0.55 TENS = MD: −0.11 CT = MD: −1.23	**0.050** 0.288 **<0.001**
		Palpation: infrahyoid area-right during voicing (4-EAI scale)	LMT = MD: −0.92 TENS = MD: −0.22 CT = MD: −1.09	**0.001** 0.126 **<0.001**
		Palpation: cricothyroid–left during voicing (4-EAI scale)	LMT = MD: −1.66 TENS = MD: −0.55 CT = MD: −1.06	**0.014** **0.021** **<0.001**
		Palpation: cricothyroid-right during voicing (4-EAI scale)	LMT = MD: −1.36 TENS = MD: −0.79 CT = MD: −1.34	**0.010** **<0.001** **0.001**
		Palpation: sternocleidomastoid muscle–left during voicing (4-EAI scale)	LMT = MD: −1.37 TENS = MD: −0.75 CT = MD: −1.48	**0.009** **0.012** **<0.001**
		Palpation: sternocleidomastoid muscle–right during voicing (4-EAI scale)	LMT = MD: −0.2.05 TENS = MD: −0.52 CT = MD: −1.14	**0.015** 0.052 **<0.001**
		Palpation: lateral movement area (4-EAI scale)	LMT = MD: −0.1.40 TENS = MD: −0.97 CT = MD: −1.01	**<0.001** **<0.001** **<0.001**
		Palpation: laryngeal high position (4-EAI scale)	LMT = MD: −1.17 TENS = MD: −0.98 CT = MD: −0.63	**<0.001** **0.007** **0.016**
Ahmadi et al. [32]	Laryngeal Manual Therapy (*n* = 14), Edge Therapy + Laryngeal Manual Therapy (*n* = 17)	Laryngeal Imaging: presence of no MTD pattern	LMT = Before: 0% LMT = After: 35.71% LMT + ET = Before: 0% LMT + ET = After: 29.4%	**<0.05** (in total of all MTD patterns)
		Laryngeal Imaging: presence of MTD pattern type 1	LMT = Before: 0% LMT = After: 14.3% LMT + ET = Before: 0% LMT + ET = After: 23.5%	
		Laryngeal Imaging: presence of MTD pattern type 2	LMT = Before: 14.3% LMT = After: 14.3% LMT + ET = Before: 0% LMT + ET = After: 5.9%	
		Laryngeal Imaging: presence of MTD pattern type 3	LMT = Before: 65.07% LMT = After: 35.71% LMT + ET = Before: 52.9% LMT + ET = After: 23.5%	
		Laryngeal Imaging: presence of MTD pattern type 4	LMT = Before: 14.3% LMT = After: 0% LMT + ET = Before: 42.2% LMT + ET = After: 11.8%	
		Laryngeal Imaging: presence of MTD pattern type 5	LMT = Before: 7.1% LMT = After: 0% LMT + ET = Before: 5.9% LMT + ET = After: 5.9%	
		Glottal Closure pattern: Complete	LMT = Before: 21.4% LMT = After: 42.9% LMT + ET = Before: 29.4% LMT + ET = After: 23.5%	**<0.05** (in total of all glottal closure patterns)
		Glottal Closure pattern: Incomplete	LMT = Before: 42.9% LMT = After: 14.3% LMT + ET = Before: 41.2% LMT + ET = After: 11.8%	
		Glottal Closure pattern: Anterior Gap	LMT = Before: 14.3% LMT = After: 14.3% LMT + ET = Before: 5.9% LMT + ET = After: 23.5%	
		Glottal Closure pattern: Posterior gap	LMT = Before: 0% LMT = After: 21.4% LMT + ET = Before: 0% LMT + ET = After: 23.5%	
		Glottal Closure pattern: Spindle gap	LMT = Before: 21.4% LMT = After: 7.1% LMT + ET = Before: 23.5% LMT + ET = After: 5.9%	
		Glottal Closure pattern: Hourglass	LMT = Before: 0% LMT = After: 0% LMT + ET = Before: 0% LMT + ET = After: 11.8%	
		F0 on vowel/a/	LMT = MD: −10.45 Hz ET + LMT = MD: −32.4 Hz	0.10 (LMT vs. ET + LMT) **0.002** (time)
		Jitter on vowel/a/	LMT = MD: −0.09% ET + LMT = MD: −0.04%	0.60 (LMT vs. ET + LMT) **<0.05** (time)
		Shimmer on vowel/a/	LMT = MD: 3.86% ET + LMT = MD: 1.91%	0.46 (LMT vs. ET + LMT) **<0.001** (time)
		HNR on vowel/a/	LMT = MD: −5.7 dB ET + LMT = MD: −3.88 dB	0.32 (LMT vs. ET + LMT) **<0.001** (time)
		SVHI *More parameters cannot be presented because of incomplete data presentation*	LMT = MeD: −14.07 ET + LMT = MeD: −31	**<0.05** **<0.001**
**Manual Circumlaryngeal Therapy (*n* = 10)**				
Roy & Tasko [33]	Manual Circumlaryngeal Therapy	F0 on continuous speech SD of F0 on continuous speech Overall perceived voice quality on a 7-EAI scale on continuous speech	MD: 3.35 Hz MD: −9.29 Hz MD: −3.7	0.888 0.098 **0.0004**
Roy [34]	Manual Circumlaryngeal Therapy	*Results are shown only for vowel/a/* F0 Jitter Shimmer SNR Overall perceived voice quality on a 7-EAI scale	D1: −21.5 ST D2: −20.3 ST D1: −0.068 ms D2: −0.05 ms D1: −5.75% D2: −13.5% D1: 0.88 dB D2: 8.09 dB D1: −5.15 D2: −6	N/A
Roy & Hendarto [35]	Manual Circumlaryngeal Therapy	F0 on continuous speech Overall perceived voice quality on a 13 cm VAS scale on continuous speech	MD: −1.91 Hz MD: −6.74 cm	0.80 **<0.0001**
Russel [36]	Manual Circumlaryngeal Therapy	*Results are shown only for vowel/a/* Overall perceived voice quality (10 cm VAS scale) Perceived breathiness (10 cm VAS scale) Perceived pitch (10 cm VAS scale) Perceived strain (10 cm VAS scale) Perceived loudness (10 cm VAS scale) F0 Jitter Shimmer	DP: −28% D3: −42% DP: −44% D3: −39% DP: −52% D3: −55% DP: −13% D3: −8% DP: −58% D3: −52% DP: −85 Hz D3: −80 Hz DP: −3.1% D3: −3.5% DP: −2.8% D3: −2.0%	**<0.001** **<0.000** **<0.000** **<0.000** **<0.000** **<0.000** **<0.002** **<0.023** **<0.000** **<0.000** **<0.0001** **<0.0001** **<0.0001** **<0.0001** **<0.0001** **<0.0004**
D’haeseleer et al. [37]	Manual Circumlaryngeal Therapy (*n* = 8), Voice Rest (*n* = 7)	MPT on vowel/a/ I-low on vowel/a/ I-high on vowel/a/ F0-low on vowel/a/ F0-high on vowel/a/ F0 on vowel/a/ Jitter on vowel/a/ Shimmer on vowel/a/ NHR on vowel/a/ DSI on vowel/a/	MCT = MeD: 2.1 s VR = MeD: −0.5 s MCT = MeD: −0.5 dB VR = MeD: 1 dB MCT = MeD: −2 dB VR = MeD: 4 dB MCT = MeD: 7.2 Hz VR = MeD: −7.8 Hz MCT = MeD: 182.6 Hz VR = MeD: −78.4 Hz MCT = MeD: 14.9 Hz VR = MeD: 8.2 Hz MCT = MeD: 0.1% VR = MeD: 0% MCT = MeD: −0.4% VR = MeD: 0.3% MCT = MeD: 0 dB VR = MeD: 0 dB MCT = MeD: 0.9 VR = MeD: −0.3	0.263 0.799 0.293 0.071 0.672 0.107 1.000 0.581 0.310 0.500 0.612 0.499 0.327 0.735 0.866 0.237 0.293 0.498 **0.050** 0.237
Roy et al. [38]	Manual Circumlaryngeal Therapy	*Results are shown only for vowel/a/* F0 CSID Overall perceived voice quality (VAS 100 mm)	MD: −51.3 Hz MD: −8.8 MD: −26.6 mm	**0.000** 0.399 **0.000**
Barsties v. Latoszek [39]	Manual Circumlaryngeal Therapy	AVQI ABI DSI on the vowel/a/ F0 on continuous speech SPL on continuous speech F0-high on the vowel/a/ F0-low on the vowel/a/ I-high on the vowel/a/ I-low on the vowel/a/ VHI-T VHI-p VHI-f VHI-e	MD-mP: −0.72 MD-mP: −1.34 MD-mP: −0.9 MD-mP: −77.04 Hz MD-mP: −3.94 dB MD-mP: −40.86 Hz MD-mP: −51.47 Hz MD-mP: 3.13 dB MD-mP: −1.25 dB MD-mP: −70.50 MD-mP: −24 MD-mP: −22.5 MD-mP: −24	N/A
Pani et al. [40]	Manual Circumlaryngeal Therapy	Overall perceived voice quality (4-EAI scale) Perceived roughness (4-EAI scale) Perceived breathiness (4-EAI scale) Perceived asthenia (4-EAI scale) Perceived strain (4-EAI scale) *More parameters cannot be presented because of incomplete or errors in data presentation to create quantitative pairwise differences*	DP: −1 DP: −3 DP: −2 DP: −3 DP: −3	N/A
Shabani et al. [41]	Manual Circumlaryngeal Therapy (*n* = 5), Manual Circumlaryngeal Therapy + Transcutaneous Electrical Nerve Stimulation (*n* = 5)	DSI	MCT = MD: 1.35 MCT + TENS = MD: 2.23	**0.012** **0.016** **0.037** (MCT vs. MCT + TENS)
		*Results are shown only for vowel/e/* EMG: suprahyoid	MCT = MD: −0.11 MCT + TENS = MD: −0.20	**0.003** **0.002** 0.170 (MCT vs. MCT + TENS)
		EMG: infrahyoid right	MCT = MD: −0.14 MCT + TENS = MD: −0.24	**0.000** **0.000** 0.257 (MCT vs. MCT + TENS)
		EMG: infrahyoid right	MCT = MD: −0.11 MCT + TENS = MD: −0.18	**0.002** **0.000** **0.02** (MCT vs. MCT + TENS)
Behroozi et al. [42]	Manual Circumlaryngeal Therapy (*n* = 20), Manual Circumlaryngeal Therapy + herbs (*n* = 20)	DSI	MCT = MD: 1.69 MCT + herbs = MD: 2.45	**<0.001** **<0.001** **0.007** (MCT vs. MCT + herbs)
		VTD-f	MCT = MD: −16.3 MCT + herbs = MD: −21.5	**0.001** **0.001** **0.01** (MCT vs. MCT + herbs)
		VTD-s	MCT = MD: −15.6 MCT + herbs = MD: −20.95	**0.001** **0.001** **0.01** (MCT vs. MCT + herbs)
		Pain (VAS 100 mm)	MCT = MD: −37 mm MCT + herbs = MD: −41 mm	**<0.05** **<0.05** 0.583 (MCT vs. MCT + herbs)
**Cricothyroid Visor Maneuver (*n* = 3)**				
Dehqan [43]	Cricothyroid Visor Maneuver	F0 on vowel/a/ Jitter on vowel/a/ Shimmer on vowel/a/ HNR on vowel/a/ CPPS on vowel/a/ MPT on vowel/a/	MD-mP: 6.71 Hz MD-mP: −0.1% MD-mP: −0.77% MD-mP: −0.17 dB MD-mP: 1.05 dB MD-mP: 0.39 s	N/A
		Overall perceived voice quality (VAS 100 mm)	DP: −42.5 mm	
		Perceived roughness (VAS 100 mm) Perceived breathiness (VAS 100 mm) Perceived strain (VAS 100 mm) Perceived pitch (VAS 100 mm) Perceived loudness (VAS 100 mm) *Results are shown only for the total score of VRQOL* VRQOL	DP: −40 mm DP: −25 mm DP: −22.5 mm DP: −25 mm DP: −30 mm DP: 49	
Moein et al. [44]	Cricothyroid Visor Maneuver	F0 on vowel/a/ Jitter on vowel/a/ Shimmer on vowel/a/ HNR on vowel/a/ CPPS on vowel/a/ MPT on vowel/a/	DP: 1.41 Hz DP: −0.065% DP: −1.26% DP: −0.15 dB DP: 3.29 dB DP: 2.11 s	N/A
		Overall perceived voice quality (VAS 100 mm)	DP: −30.5 mm	
		Perceived roughness (VAS 100 mm) Perceived breathiness (VAS 100 mm) Perceived strain (VAS 100 mm) Perceived pitch (VAS 100 mm) Perceived loudness (VAS 100 mm) Self-evaluated voice quality (VAS 100 mm) *More parameters cannot be presented because of incomplete data presentation*	DP: −19.5 mm DP: −10.5 mm DP: −22.5 mm DP: −23.5 mm DP: −11 mm DP: 50 mm	
Nasrin et al. [45]	Cricothyroid Visor Maneuver	F0 (%-change) on vowel/a/ Jitter on vowel/a/ Shimmer on vowel/a/ HNR on vowel/a/ CPPS on vowel/a/ DSI on vowel/a/ MPT on vowel/a/	DP: 2.8% MD: −0.63% MD: −0.11% MD: 1.44 dB MD: 5.01 dB MD: 1.05 MD: 3.87 s	0.753 0.116 0.753 0.600 **0.028** 0.173 **0.028**
		Overall perceived voice quality (VAS 100 mm)	MD: −34.67 mm	**0.027**
		VRQOL VTD-f VTD-s	MD: 47.5 MD: −25 MD: −28.5	**0.026** **0.027** **0.026**
**Voice Massage (*n* = 3)**				
Laukkanen et al. [46]	Voice Massage (*n* = 30), Voice Training (*n* = 30), a single Vocal Hygiene approach (*n* = 30)	Self-perceived positive voice influence on VAS 100 mm after therapy *More parameters cannot be presented because of lack of quantitative data presentation by Laukkanen et al.* [46]	VM = MD: 66.6 mm VT = MD: 56.2 mm VH = MD: 27.5 mm	**0.000** (VM vs. VH) **0.000** (VT vs. VH)
Leppänen et al. [47]	Voice Massage (*n* = 30), Vocal Hygiene (*n* = 29),	*Results are shown only for vowel/a/on habitual voice*		
		F0, beginning of term	VM = MD-MvsA: 5.5 Hz VH = MD-MvsA: 11 Hz	>0.05 **0.031**
		F0, end of term	VM = MD-MvsA: 4.4 Hz VH = MD-MvsA: 4.5 Hz Begin vs. end: differences in mean values and changes	>0.05 >0.05 >0.05
		Leq, beginning of term	VM = MD-MvsA: 0.8 dB VH = MD-MvsA: 2.1 dB	>0.05 **0.045**
		Leq, end of term	VM = MD-MvsA: −0.2 dB VH = MD-MvsA: 2.7 dB Begin vs. end: differences in mean values and changes	>0.05 **0.01** >0.05
		SD of jitter, beginning of term	VM = MD-MvsA: −0.039% VH = MD-MvsA: −0.034%	>0.05 >0.05
		SD of jitter, end of term	VM = MD-MvsA: 0.005% VH = MD-MvsA: −0.003% Begin vs. end: differences in mean values and changes	>0.05 >0.05 >0.05
		Jitter, beginning of term	VM = MD-MvsA: −0.159% VH = MD-MvsA: −0.096%	**0.049** >0.05
		Jitter, end of term	VM = MD-MvsA: −0.078% VH = MD-MvsA: −0.047% Begin vs. end: differences in mean values and changes	>0.05 >0.05 >0.05
		Shimmer, beginning of term	VM = MD-MvsA: −0.04 dB VH = MD-MvsA: −0.16 dB	>0.05 **0.028**
		Shimmer, end of term	VM = MD-MvsA: −0.039 dB VH = MD-MvsA: −0.055 dB Begin vs. end: differences in mean values and changes	>0.05 >0.05 >0.05
		*Results are only shown for habitual reading*		
		Perceptual strain (VAS 100 mm), beginning of term	VM = MD-MvsA: 5.53 mm VH = MD-MvsA: 9.12 mm	**0.039** **0.013**
		Perceptual strain (VAS 100 mm), end of term	VM = MD-MvsA: 8.57 mm VH = MD-MvsA: 7.6 mm Beginning vs. end: differences in mean values and changes	**0.006** **0.004** >0.05
		Perceptual breathy (VAS 100 mm), beginning of term	VM = MD-MvsA: −6.17 mm VH = MD-MvsA: −6.75 mm	**0.003** **0.010**
		Perceptual breathy (VAS 100 mm), end of term	VM = MD-MvsA: −5.07 mm VH = MD-MvsA: −8.25 mm Begin vs. end: differences in mean values and changes	**0.006** **0.002** >0.05
		Perceptual hoarse (VAS 100 mm), beginning of term	VM = MD-MvsA: −4.47 mm VH = MD-MvsA: −5.21 mm	**0.020** **0.034**
		Perceptual hoarse (VAS 100 mm), end of term	VM = MD-MvsA: −5.07 mm VH = MD-MvsA: −2.38 mm beginning vs. end: differences in mean values and changes	**0.021** >0.05 >0.05
		Self-evaluated difficulty of voice production (VAS 200 mm), beginning of term	VM = MD-MvsA: 6.37 mm VH = MD-MvsA: 6.80 mm	>0.05 >0.05
		Self-evaluated difficulty of voice production (VAS 200 mm), end of term	VM = MD-MvsA: 5.94 mm VH = MD-MvsA: −2.30 mm Beginning vs. end: differences in mean values only for VH	>0.05 >0.05 **0.007**
		Self-evaluated voice quality (VAS 200 mm), beginning of term	VM = MD-MvsA: 3.07 mm VH = MD-MvsA: 1.93 mm	>0.05 >0.05
		Self-evaluated voice quality (VAS 200 mm), end of term	VM = MD-MvsA: −3.74 mm VH = MD-MvsA: 1.96 mm Begin vs. end: differences in mean values and changes	>0.05 >0.05 >0.05
		Self-evaluated tiredness of throat (VAS 100 mm), beginning of term	VM = MD-MvsA: 12.87 mm VH = MD-MvsA: 8.83 mm	**0.033** **0.002**
		Self-evaluated tiredness of throat (VAS 100 mm), end of term	VM = MD-MvsA: 12.03 mm VH = MD-MvsA: 7.00 mm beginning vs. end: differences in mean values and changes	**0.013** >0.05 >0.05
Leppänen et al. [48]	Voice Massage (*n* = 19), Voice Training (*n* = 17), a single Vocal Hygiene approach (*n* = 13)	*Results are shown only after 1-year follow-up in comparison before intervention* Voice symptom scale (ranging from 0 to 60)	VM = MD: −7.6 VT = MD: −5.6 VH = MD: −5.7	0.946 (VM vs. VH vs. VT)
**Larynx Manipulation (*n* = 2)**				
Van Rijsen [49]	Larynx Manipulation	Palpation scale (ranging from 0 to 18) *More parameters cannot be presented because of lack of quantitative data presentation by Rijsen* [49]	DP: −12	N/A
Ahmadi et al. [50]	Larynx Manipulation (*n* = 15), Breathing exercises (*n* = 11), combined therapy (*n* = 13), and a control group who received no therapy (*n* = 12)	*Results are shown only for vowel/a/* MPT	LM = MD: 3.33 s BE = MD: 7.91 s CT = MD: 4.93 s CG = MD: 0.84 s	**0.34** (among all groups before treatment) **0.01** (among all groups after treatment)
		SVHI	LM = MD: −3.67 BE = MD: −12.64 CT = MD: −25.53 CG = MD: 2.08	0.36 (among all groups before treatment) **0.001** (among all groups after treatment)
		*More parameters cannot be presented because of incomplete data presentation*		
**Vanderbilt Manual Intervention (*n* = 2)**				
Tomlinson & Archer [51]	Vanderbilt Manual Intervention	VHI-T VHI-p VHI-f VHI-e PSFS (11-EAI scale) Pain (10-EAI scale) ROM-CLF-R ROM-CLF-L ROM-CF ROM-CE ROM-CR-R ROM-CR-L Jaw opening	MD: −14 MD: −5.14 MD: −4.29 MD: −4.57 MD: 3.33 MD: −1.22 MD: 8.33° MD: 8.89° MD: 12.22° MD: 19.44° MD: 13.89° MD: 13.89° MD: 10.78 mm	N/A
Craig et al. [52]	Vanderbilt Manual Intervention (*n* = 16), Voice therapy (*n* = 33), combined therapy (*n* = 16), none/incomplete (*n* = 88)	VHI for participants with mild dysphonia	VMI = MeD: −6.5 VT = MeD: −3.5 CT = MeD: −3 None = MeD: −0.5	0.37
		VHI for participants with moderate to severe dysphonia	VMI = MeD: −10 VT = MeD: −10 CT = MeD: −8 None = MeD: −2	**0.02** (VT vs. none) 0.99 (PT vs. VT vs. CT)
**Others (*n* = 1)**				
Sullivan et al. [53]	Traditional Chinese Massage	F0 on vowel/a/ Self-evaluated muscle tension in the laryngeal musculature (VAS 100 mm)	DPP: −44.45 Hz DPP: −37.8%	N/A
Ternström et al. [54]	Body Massage (*n* = 16), Lying on Bed (*n* = 15)	F0 on continuous speech SPL on continuous speech Area of the speech range profile	BM = MD: −1.0 ST L-B = MD: 0.1 ST BM = MD: −1.2 dB L-B = MD: −0.2 dB BM = MD: −10.31 ST/dB L-B = MD: −5.47 ST/dB	**0.0007** 0.631 **0.00007** 0.530 0.051 0.059
Voerman et al. [55]	Laryngeal Shaking (*n* = 38), Mental Imaginary (*n* = 54), Combined Therapy (*n* = 24)	Self-evaluated retrieving normal voice (after treatment) Self-evaluated relapse for participants who received more than 1 relapse (*n* = 30%)	LS: 71% MI: 96% CT: 63% LS: 15% MI: 54% CT: 31%	N/A
Mumović [56]	Laryngeal Compression (*n* = 33), Standard voice therapy (*n* = 33)	Overall perceived voice quality (4-EAI scale) Perceived instability (4-EAI scale) Perceived roughness (4-EAI scale) Perceived breathiness (4-EAI scale) Perceived asthenia (4-EAI scale) Perceived strain (4-EAI scale) F0 (modus) on vowel/a/ F0 (mean) on vowel/a/ F0-SD on vowel/a/ F0 (maximum-habitual) on vowel/a/ F0 (minimum-habitual) on vowel/a/ I-max (habitual) on vowel/a/ I-min (habitual) on vowel/a/ Jitter Shimmer HNR NNE SNR	LC = MD: −0.59 SVT = MD: −0.68 LC = MD: −0.62 SVT = MD: −0.62 LC = MD: −0.58 SVT = MD: −0.53 LC = MD: −0.87 SVT = MD: −0.78 LC = MD: −1.14 SVT = MD: −0.81 LC = MD: −0.61 SVT = MD: −0.71 LC = MD: −20.27 Hz SVT = MD: −0.12 Hz LC = MD: −20.91 Hz SVT = MD: 1.2 Hz LC = MD: −2.61 Hz SVT = MD: −1.93 Hz LC = MD: −19.26 Hz SVT = MD: −1.82 Hz LC = MD: −12.21 Hz SVT = MD: 3.46 Hz LC = MD: −0.51 dB SVT = MD: 0.21 dB LC = MD: −0.72 dB SVT = MD: 1.21 dB LC = MD: −0.53% SVT = MD: −0.26% LC = MD: −0.57% SVT = MD: −1.57% LC = MD: 2.75 dB SVT = MD: 0.95 dB LC = MD: −0.84 dB SVT = MD: −0.64 dB LC = MD: 1.97 dB SVT = MD: 1.03 dB	**0.000** **0.000** **0.000** **0.000** **0.000** **0.000** **0.000** **0.000** **0.000** **0.000** **0.000** **0.000** **0.013** >0.05 **0.008** >0.05 **0.012** >0.05 **0.039** >0.05 >0.05 >0.05 >0.05 >0.05 >0.05 >0.05 **0.001** **0.003** >0.05 >0.05 **0.009** >0.05 **0.001** **0.035** **0.032** >0.05
Flock & King [57]	Vocal Manual Therapy Intervention	RSI (summarized over the 4 therapy sessions)	MD: −8.63	N/A
de Brito et al. [58]	*All participants:* Physiotherapeutic intervention/three Neural Mobilization Techniques by American Osteopathic Association vs. Voice Rest	*Results are shown only for vowel/a/* F0 Jitter PPQ Shimmer APQ NHR CPP CPPs	NMT = MD: −3.27 Hz VR = MD: −5.31 Hz NMT = MD: 0.24% VR = MD: 0.31% NMT = MD: 0.19% VR = MD: 0.20% NMT = MD: −0.07% VR = MD: 0.19% NMT = MD: −0.12% VR = MD: 0.04% NMT = MD: 0.00 dB VR = MD: 0.00 dB NMT = MD: −0.63 dB VR = MD: −0.37 dB NMT = MD: −0.78 dB VR = MD: −0.48 dB	0.943 (NMT vs. VR vs. baseline) 0.229 (NMT vs. VR vs. baseline) 0.131 (NMT vs. VR vs. baseline) 0.717 (NMT vs. VR vs. baseline) 0.827 (NMT vs. VR vs. baseline) 0.322 (NMT vs. VR vs. baseline) 0.748 (NMT vs. VR vs. baseline) 0.506 (NMT vs. VR vs. baseline) 0.283 (NMT vs. VR)
		Overall perceived voice quality (pairwise rating to baseline)	NMT = improved: 5% NMT = worsened: 0% NMT = unchanged: 95% VR = improved: 0% VR = worsened: 14% VR = unchanged: 86%	
		Self-perceived vocal effort (VAS 10 cm)	NMT = MD: 1.66 cm VR = MD: 4.01 cm	**0.004** (NMT vs. VR)
		Palpation: Supralaryngeal resistance (range: 1–5, in comparison to baseline)	NMT = MD: −0.29 VR = MD: −0.10	0.615 (VR vs. baseline) 0.898 (NMT vs. VR)
		Palpation: Larynx Lateral Resistance (range: 1–5, in comparison to baseline)	NMT = MD: −0.43 VR = MD: −0.04	0.987 (VR vs. baseline) 0.524 (NMT vs. VR)
		Palpation: Larynx Position	Baseline = Neutral: 62% Elevated: 38% NMT = Neutral: 76% Elevated: 24% VR = Neutral: 67% Elevated: 33%	987 (VR vs. baseline) 0.524 (NMT vs. VR)

***Abbreviation*:** ABI = Acoustic Breathiness Index; AP-compression = Anteroposterior compression; APQ = Amplitude Perturbation Quotient; AVQI = Acoustic Voice Quality Index; BE = Breathing Exercises; BM = Body Massage; CG = Control Group; CPP = Cepstral Peak Prominence; CPPs = Smoothed Cepstral Peak Prominence; CSID = Cepstral Spectral Index of Dysphonia; CT = Combined Therapy; DDCavR = Average diadochokinesis rate; DDCavP = Repetition rate of diadochokinesis; DDCdpP = Standard deviation of diadochokinesis period; DDCcvP = Diadochokinesis period variation coefficient; DDCjiitP = Disturbances of diadochokinesis period; DDCcvI = Diadochokinesis intensity peak variation coefficient; D1 = Difference after 1 Month in comparison with the baseline measurement; D2 = Difference after 2 Month in comparison with the baseline; D3 = Difference after 1 year in comparison with the baseline; DP = Difference between pretreatment and posttreatment without mean values (case study); DPP = Difference between more pretreatment assessments (combined as mean result) and posttreatment (also possible to have more short-time follow-up assessments after treatment, then also combined as mean result); DSI = Dysphonia Severity Index; EAI scale = Equal-Appearing Interval Scale; EMG = Electromyography; ET = Edge Therapy; F0 = Fundamental Frequency; F0-high = highest Fundamental Frequency; F0-low = lowest fundamental frequency; HNR = Harmonic-to-Noise Ratio; Hz = Hertz; I-high = highest intensity; I-low = lowest intensity; L-B = lying on bed; LC = Laryngeal Compression; Leq = an energy mean of the sound level averaged over the measurement period; LS = Laryngeal Shaking; LMT = Laryngeal Manual Therapy; MCT = Manual Circumlaryngeal Therapy; MD = Mean Difference of pre–post measurement; MeD = Median Difference; MD-MvsA = Mean difference between morning vs. afternoon assessment of a working day; MD-mP = Mean difference between a pre-assessment and more post-treatment assessments depending on number of therapy sessions; MI = Mental Imaginary; ML-compression = Mediolateral compression; MPI = Musculoskeletal Pain Investigation; MPT = Maximum Phonation Time; MTD pattern = Muscle Tension Dysphonia pattern in which type 1: laryngeal isometric MTD, type 2: medial hyperadduction, type 3: anteroposterior constriction of the supraglottic larynx, type 4: hypoadducted vocal folds, type 5: psychogenic dysphonia; NHR = Noise-to-Harmonics Ratio; NMT = Neural Mobilization Techniques; NNE = normalized noise energy; PPQ = Period Perturbation Quotient; PSFS = Patient-Specific Functional Scale; ROM = Range of Motion; ROM-CLF-R = Range of Motion of Cervical Lateral Flexion, right; ROM-CLF-L = Range of Motion of Cervical Lateral Flexion, left; ROM-CF = Range of Motion of Cervical Flexion; ROM-CE = Range of Motion of Cervical Extension; ROM-CR-R = Range of Motion of Cervical Rotation, right; ROM-CR-L = Range of Motion of Cervical Rotation, left; RSI = Reflux Symptom Index; SD = Standard Deviation; SNR = Signal-to-Noise-Ratio; SPL = Sound Pressure Level; ST = Semitones; SVHI = Singing Voice Handicap Index; SVT = Standard Voice Therapy; S/Z Ratio = ratio of maximum sustained time for a speaker to produce both/*S*/and/*Z*/sounds; TENS = Transcutaneous Electrical Nerve Stimulation; VAS = Visual Analogue Scale; VFI = Vocal Fatigue Index; VH = Vocal Hygiene; VHI-T = Voice Handicap Index total score; VHI-p = physical subscale of the Voice Handicap Index; VHI-f = functional subscale of the Voice Handicap Index; VHI-e = emotional subscale of the Voice Handicap Index; VM = Voice Massage; VMI = Vanderbilt Manual Intervention; VR = Voice Rest; VRQOL = Voice-Related Quality of Life; VSS = Vocal Symptoms Scale; VT = Voice Training; VTD-f = Vocal Tract Discomfort scale Frequency subscale; VTD-s = Vocal Tract Discomfort scale Severity subscale. Bold numbers indicate statistically significant results at *p* ≤ 0.05.

**Table 4:** Results from meta-analysis.

Study	Group of Comparison	Outcome Measure	Effect Estimation	Level of Certainty of Evidence
Ribeiro et al. [59]	LMT vs. other direct voice therapy approaches (e.g., TENS and vocal training composed of the tongue or lip trill, humming, overarticulation, and chewing techniques)	Perceived overall voice quality	RR: 1.63 95% CI: 0.53 to 5.02	N/A
		Self-evaluated intensity of laryngeal symptoms	RR: 1.28 95% CI: 0.80 to 2.04	
		Self-evaluated intensity of musculoskeletal pain	RR: 0.58 95% CI: 0.30 to 1.11	
Kim [60]	Cumulative manual laryngeal therapy of MCT (*n* = 9), MCT + CVM (*n* = 1), LMT (*n* = 1)	Jitter	Hedges *g*: −0.58 95% CI: −0.85 to −0.31 *p*-value: **<0.001**	N/A
		Shimmer	Hedges *g*: −0.39 95% CI: −0.69 to −0.09 *p*-value: **0.010**	
		HNR/NHR	Hedges *g*: 0.60 95% CI: 0.22 to 0.98 *p*-value: **0.002**	
		F0-high	Hedges *g*: 0.73 95% CI: −0.49 to −1.94 *p*-value: 0.242	
		I-low	Hedges *g*: −1.07 95% CI: −1.67 to −0.47 *p*-value: **0.001**	
		DSI	Hedges *g*: 2.03 95% CI: 1.23 to 2.83 *p*-value: **<0.001**	
		CPPs	Hedges *g*: 0.77 95% CI: 0.01 to 1.53 *p*-value: **0.048**	
		F1	Hedges *g*: −0.71 95% CI: −1.17 to −0.26 *p*-value: **0.002**	
		F2	Hedges *g*: −0.80 95% CI: −1.31 to −0.28 *p*-value: **0.002**	
		F1-slope	Hedges *g*: 0.12 95% CI: −0.08 to 0.33 *p*-value: 0.242	
		F2-slope	Hedges *g*: 0.37 95% CI: 0.23 to 0.50 *p*-value: **<0.001**	
Barsties v. Latoszek et al. [61]	MCT	F0	MD: −5.18 Hz 95% CI: −20.82 to 10.46 Cohen’s *d*: 1.20	N/A
		Jitter	MD: −0.58% 95% CI: **−1.00 to −0.16** Cohen’s *d*: 2.64	
		Shimmer	MD: −5.66% 95% CI: **−8.16 to −3.17** Cohen’s *d*: 4.24	
		HNR	MD: 4.65 dB 95% CI: **1.90 to 7.41** Cohen’s *d*: 4.93	
Barsties v. Latoszek et al. [62]	MCT (*n* = 1) vs. control group with, no treatment or placebo (*n* = 8) in a network meta-analysis (considering also 23 other voice treatments)	Perceived overall voice quality (0 = normal to 3 = severe)	MD: −1.50 95% CI: **−2.36 to −0.63**	Very low

*Abbreviation*: RR = Relative Risk Ratio; CI = Confidence Interval; MCT = Manual Circumlaryngeal Therapy; CVM = Cricothyroid Visor Maneuver; LMT = Laryngeal Manual Therapy; HNR = Harmonic-to-Noise Ratio; NHR = Noise-to-Harmonics Ratio; F0 = Fundamental Frequency; F0-high = highest Fundamental Frequency; I-low = lowest intensity; DSI = Dysphonia Severity Index; CPPs = Smoothed Cepstral Peak Prominence; F1 = first Formant; F2 = second Formant; F1-slope = slope of the first Formant; F2-slope = slope of the second Formant; TENS = Transcutaneous Electrical Nerve Stimulation. Bold numbers indicate statistically significant results at *p* ≤ 0.05.

## Data Availability

The original contributions presented in the study are included in the article; further inquiries can be directed to the corresponding author.

## Supplementary Materials

The following supporting information can be downloaded at: https://www.mdpi.com/article/10.3390/jcm15218671/s1, Table S1: Preferred Reporting Items for Systematic reviews and Meta-Analyses extension for Scoping Reviews (PRISMA-ScR) Checklist; Table S2: Search strategies for systematic review; Table S3: List of excluded papers during the eligibility process.
