# Supplementary material for: Manual Laryngeal Therapy Across Clinical Populations: A Scoping Review
Source: J Clin Med. 2026 Nov 6;15(21):8671. doi: 10.3390/jcm15218671 (PMC13763639; doi:10.3390/jcm15218671)
Supplement: Supplementary file 1 [file jcm-15-08671-s001.zip › jcm-4636086-supplementary.pdf]

Table S1: Preferred Reporting Items for Systematic reviews and Meta-Analyses extension for Scoping Reviews (PRISMA-ScR) Checklist

SECTION	ITEM	PRISMA-ScR CHECKLIST ITEM	REPORTED ON PAGE #
**TITLE**			
Title	1	Identify the report as a scoping review.	1
**ABSTRACT**			
Structured summary	2	Provide a structured summary that includes (as applicable): background, objectives, eligibility criteria, sources of evidence, charting methods, results, and conclusions that relate to the review questions and objectives.	1
**INTRODUCTION**			
Rationale	3	Describe the rationale for the review in the context of what is already known. Explain why the review questions/objectives lend themselves to a scoping review approach.	2
Objectives	4	Provide an explicit statement of the questions and objectives being addressed with reference to their key elements (e.g., population or participants, concepts, and context) or other relevant key elements used to conceptualize the review questions and/or objectives.	2
**METHODS**			
Protocol and registration	5	Indicate whether a review protocol exists; state if and where it can be accessed (e.g., a Web address); and if available, provide registration information, including the registration number.	2
Eligibility criteria	6	Specify characteristics of the sources of evidence used as eligibility criteria (e.g., years considered, language, and publication status), and provide a rationale.	3 & 4
Information sources*	7	Describe all information sources in the search (e.g., databases with dates of coverage and contact with authors to identify additional sources), as well as the date the most recent search was executed.	3
Search	8	Present the full electronic search strategy for at least 1 database, including any limits used, such that it could be repeated.	Table S2
Selection of sources of evidence†	9	State the process for selecting sources of evidence (i.e., screening and eligibility) included in the scoping review.	3 & 4
Data charting process‡	10	Describe the methods of charting data from the included sources of evidence (e.g., calibrated forms or forms that have been tested by the team before their use, and whether data charting was done independently or in duplicate) and any processes for obtaining and confirming data from investigators.	4
Data items	11	List and define all variables for which data were sought and any assumptions and simplifications made.	3 & 4
Critical appraisal of individual sources of evidence§	12	If done, provide a rationale for conducting a critical appraisal of included sources of evidence; describe the methods used and how this information was used in any data synthesis (if appropriate).	4
Synthesis of results	13	Describe the methods of handling and summarizing the data that were charted.	4

SECTION	ITEM	PRISMA-ScR CHECKLIST ITEM	REPORTED ON PAGE #
**RESULTS**			
Selection of sources of evidence	14	Give numbers of sources of evidence screened, assessed for eligibility, and included in the review, with reasons for exclusions at each stage, ideally using a flow diagram.	4, Table S3, Figure 1
Characteristics of sources of evidence	15	For each source of evidence, present characteristics for which data were charted and provide the citations.	Tables 1 and 2
Critical appraisal within sources of evidence	16	If done, present data on critical appraisal of included sources of evidence (see item 12).	Table 3
Results of individual sources of evidence	17	For each included source of evidence, present the relevant data that were charted that relate to the review questions and objectives.	Tables 3 and 4
Synthesis of results	18	Summarize and/or present the charting results as they relate to the review questions and objectives.	5 to 7
**DISCUSSION**			
Summary of evidence	19	Summarize the main results (including an overview of concepts, themes, and types of evidence available), link to the review questions and objectives, and consider the relevance to key groups.	34 to 37
Limitations	20	Discuss the limitations of the scoping review process.	36 & 37
Conclusions	21	Provide a general interpretation of the results with respect to the review questions and objectives, as well as potential implications and/or next steps.	37
**FUNDING**			
Funding	22	Describe sources of funding for the included sources of evidence, as well as sources of funding for the scoping review. Describe the role of the funders of the scoping review.	37

JBI = Joanna Briggs Institute; PRISMA-ScR = Preferred Reporting Items for Systematic reviews and Meta-Analyses extension for Scoping Reviews.
* Where *sources of evidence* (see second footnote) are compiled from, such as bibliographic databases, social media platforms, and Web sites.
† A more inclusive/heterogeneous term used to account for the different types of evidence or data sources (e.g., quantitative and/or qualitative research, expert opinion, and policy documents) that may be eligible in a scoping review as opposed to only studies. This is not to be confused with *information sources* (see first footnote).
‡ The frameworks by Arksey and O'Malley (6) and Levac and colleagues (7) and the JBI guidance (4, 5) refer to the process of data extraction in a scoping review as data charting.
§ The process of systematically examining research evidence to assess its validity, results, and relevance before using it to inform a decision. This term is used for items 12 and 19 instead of "risk of bias" (which is more applicable to systematic reviews of interventions) to include and acknowledge the various sources of evidence that may be used in a scoping review (e.g., quantitative and/or qualitative research, expert opinion, and policy document).

From: Tricco AC, Lillie E, Zarin W, O'Brien KK, Colquhoun H, Levac D, et al. PRISMA Extension for Scoping Reviews (PRISMAScR): Checklist and Explanation. Ann Intern Med. 2018;169:467–473. doi: 10.7326/M18-0850.

Table S2: Search strategies for systematic review

Database	Search strategy	Number of Records
PubMed	(“manual circumlaryngeal therapy”[tiab] OR “circumlaryngeal manual therapy”[tiab] OR “laryngeal manual therapy”[tiab] OR “laryngeal manual voice therapy”[tiab] OR “manual laryngeal musculoskeletal tension reduction”[tiab] OR “laryngeal musculoskeletal tension reduction”[tiab] OR “laryngeal tension reduction”[tiab] OR “laryngeal shaking”[tiab] OR “voice massage”[tiab] OR “laryngeal massage”[tiab] OR “perilaryngeal massage”[tiab] OR “perilaryngeal manual massage”[tiab] OR “circumlaryngeal massage”[tiab] OR “circumlaryngeal manipulation”[tiab] OR “laryngeal reposturing”[tiab] OR “laryngeal relaxation”[tiab])<br>OR<br>(<br>(voice[Mesh] OR larynx [Mesh] OR deglutition[Mesh] OR dysphonia[Mesh] OR laryn*[tiab] OR perilaryn*[tiab] OR circumlaryn*[tiab] OR voice[tiab] OR vocal[tiab] OR dysphon*[tiab] OR dysphag*[tiab] OR swallow*[tiab])<br>AND<br>(massage[Mesh] OR “musculoskeletal manipulations”[Mesh] OR relaxation[Mesh] OR “muscle relaxation”[Mesh] OR massage[tiab] OR “manual therapy”[tiab] OR “manual treatment”[tiab] OR manipulation*[tiab] OR “musculoskeletal tension reduction”[tiab] OR relaxation[tiab])<br>)	4,282
CINAHL Complete (via EBSCO)	(XB (manual circumlaryngeal therapy) OR XB (circumlaryngeal manual therapy) OR XB (laryngeal manual therapy) OR XB (laryngeal manual voice therapy) OR XB (manual laryngeal musculoskeletal tension reduction) OR XB (laryngeal musculoskeletal tension reduction) OR XB (laryngeal tension reduction) OR XB (laryngeal shaking) OR XB (voice massage) OR XB (laryngeal massage) OR XB (perilaryngeal massage) OR XB (perilaryngeal manual massage) OR XB (circumlaryngeal massage) OR XB (circumlaryngeal manipulation) OR XB (laryngeal reposturing) OR XB (laryngeal relaxation))	938
APA PsychINFO (via EBSCO)	(XB (manual circumlaryngeal therapy) OR XB (circumlaryngeal manual therapy) OR XB (laryngeal manual therapy) OR XB (laryngeal manual voice therapy) OR XB (manual laryngeal musculoskeletal tension reduction) OR XB (laryngeal musculoskeletal tension reduction) OR XB (laryngeal tension reduction) OR XB (laryngeal shaking) OR XB (voice massage) OR XB (laryngeal massage) OR XB (perilaryngeal massage) OR	937

	XB (perilaryngeal manual massage) OR XB (circumlaryngeal massage) OR XB (circumlaryngeal manipulation) OR XB (laryngeal reposturing) OR XB (laryngeal relaxation))<br>OR<br>(<br>(SU (larynx) OR SU (voice) OR SU (dysphonia) OR SU (swallowing) OR SU (dysphagia) OR XB (laryn*) OR XB (perilaryn*) OR XB (circumlaryn*) OR XB (voice) OR XB (vocal) OR XB (dysphon*) OR XB (dysphag*) OR XB (swallow*))<br>AND<br>(SU (massage) OR SU (relaxation) OR SU (relaxation therapy) OR SU (muscle relaxation) OR XB (massage) OR XB (manual therapy) OR XB (manual treatment) OR XB (manipulation) OR XB (musculoskeletal tension reduction) OR XB (relaxation))<br>)	
Embase	(‘manual circumlaryngeal therapy’:ab,ti OR ‘circumlaryngeal manual therapy’:ab,ti OR ‘laryngeal manual therapy’:ab,ti OR ‘laryngeal manual voice therapy’:ab,ti OR ‘manual laryngeal musculoskeletal tension reduction’:ab,ti OR ‘laryngeal musculoskeletal tension reduction’:ab,ti OR ‘laryngeal tension reduction’:ab,ti OR ‘laryngeal shaking’:ab,ti OR ‘voice massage’:ab,ti OR ‘laryngeal massage’:ab,ti OR ‘perilaryngeal massage’:ab,ti OR ‘perilaryngeal manual massage’:ab,ti OR ‘circumlaryngeal massage’:ab,ti OR ‘circumlaryngeal manipulation’:ab,ti OR ‘laryngeal reposturing’:ab,ti OR ‘laryngeal relaxation’:ab,ti)<br>OR<br>(<br>(‘voice’/exp OR ‘larynx’/exp OR ‘dysphonia’/exp OR ‘voice disorder’/exp OR ‘swallowing’/exp OR ‘dysphagia’/exp OR ‘laryn*’:ab,ti OR ‘perilaryn*’:ab,ti OR ‘circumlaryn*’:ab,ti OR ‘voice’:ab,ti OR ‘vocal’:ab,ti OR ‘dysphon*’:ab,ti OR ‘dysphag*’:ab,ti OR ‘swallow*’:ab,ti)<br>AND<br>(‘massage’/exp OR ‘manipulative medicine’/exp OR ‘massage’:ab,ti OR ‘manual therapy’:ab,ti OR ‘manual treatment’:ab,ti OR ‘manipulation’:ab,ti OR ‘musculoskeletal tension reduction’:ab,ti OR ‘relaxation’:ab,ti)<br>)	7,654
Web of Science	(TS=(manual circumlaryngeal therapy) OR TS=(circumlaryngeal manual therapy) OR TS=(laryngeal manual therapy) OR TS=(laryngeal manual voice therapy) OR TS=(manual laryngeal musculoskeletal tension reduction) OR TS=(laryngeal musculoskeletal tension reduction) OR TS=(laryngeal tension reduction) OR TS=(laryngeal shaking) OR TS=(voice massage) OR TS=(laryngeal massage) OR TS=(perilaryngeal massage) OR TS=(perilaryngeal manual massage) OR TS=(circumlaryngeal massage) OR	4,806

	TS=(circumlaryngeal manipulation) OR TS=(laryngeal reposturing) OR TS=(laryngeal relaxation))<br>OR<br>(<br>(TS= (larynx) OR TS=(perilaryn*) OR TS=(circumlaryn*) OR TS=(voice) OR TS=(vocal) OR TS=(dysphon*) OR TS=(dysphag*) OR TS=(swallow))<br>AND<br>(TS= (massage) OR TS=(manual therapy) OR TS=(manual treatment) OR TS=(manipulation) OR TS=(musculoskeletal tension reduction) OR TS=(relaxation))<br>)	
Ovid Emcare	(manual circumlaryngeal therapy OR circumlaryngeal manual therapy OR laryngeal manual therapy OR laryngeal manual voice therapy OR manual laryngeal musculoskeletal tension reduction OR laryngeal musculoskeletal tension reduction OR laryngeal tension reduction OR laryngeal shaking OR voice massage OR laryngeal massage OR perilaryngeal massage OR perilaryngeal manual massage OR circumlaryngeal massage OR circumlaryngeal manipulation OR laryngeal reposturing OR laryngeal relaxation). ti,ab.<br>OR<br>(<br>(exp voice/ OR exp larynx/ OR exp dysphonia/ OR exp voice disorder/ OR exp swallowing/ OR exp dysphagia/)<br>OR (laryn* OR perilaryn* OR circumlaryn* OR voice OR vocal OR dysphon* OR dysphag* OR swallow*). ti,ab.<br>)<br>AND<br>(<br>(exp massage/ OR exp manipulative medicine/)<br>OR (massage OR manual therapy OR manual treatment OR manipulation OR musculoskeletal tension reduction OR relaxation). ti,ab.<br>)	1,837
Google Scholar	"manual circumlaryngeal therapy" OR "circumlaryngeal manual therapy" OR "laryngeal manual therapy" OR "laryngeal manual voice therapy" OR "manual laryngeal musculoskeletal tension reduction" OR "laryngeal musculoskeletal tension reduction" OR "laryngeal tension reduction" OR "laryngeal shaking" OR "voice massage" OR "laryngeal massage" OR "perilaryngeal massage" OR "perilaryngeal manual massage" OR "circumlaryngeal massage" OR "circumlaryngeal manipulation"	100
Total	N/A	20,554

Table S3: List of excluded papers during the eligibility process

Reason Number from PRISMA Flow Chart	Article
#1	Fourteen articles were included in the meta-analysis and, therefore, were not reported solely as empirical trials in this review.<br><br>• Khoddami SM, Aghadoost S, Jalaie S, Dabirmoghaddam P. The comparison between vocal facilitating techniques, manual circumlaryngeal therapy, and combined voice therapy in teachers with muscle tension dysphonia: a randomized clinical trial. *Eur Arch Otorhinolaryngol*. 2023;280(10):4543-4553. doi:10.1007/s00405-023-08042-5<br>• Dehqan A, Ballard KJ. An evaluation of Short-term Treatment Outcomes of Cricothyroid Visor Maneuver: A Proof-of-Concept Pilot Study. *J Voice*. 2021;35(2):330.e1-330.e7. doi:10.1016/j.jvoice.2019.09.016<br>• Aghadoost S, Jalaie S, Khatoonabadi AR, Dabirmoghaddam P, Khoddami SM. A Study of Vocal Facilitating Techniques Compared to Manual Circumlaryngeal Therapy in Teachers With Muscle Tension Dysphonia. *J Voice*. 2020;34(6):963.e11-963.e21. doi:10.1016/j.jvoice.2019.06.002<br>• Dehqan A, Scherer RC. Positive Effects of Manual Circumlaryngeal Therapy in the Treatment of Muscle Tension Dysphonia (MTD): Long Term Treatment Outcomes. *J Voice*. 2019;33(6):866-871. doi:10.1016/j.jvoice.2018.07.010<br>• Rad AR, Moradi N, Shater Zadeh Yazdi MJ, Soltani M, Mehravar M, Latifi SM. Efficacy of manual circumlaryngeal therapy in patients with muscle tension dysphonia. *Shiraz E-Med J*. 2018;19(7):e64478.<br>• Alves Silverio KC, Brasolotto AG, Thaís Donalonso Siqueira L, Carneiro CG, Fukushiro AP, Roberto de Jesus Guirro R. Effect of application of transcutaneous electrical nerve stimulation and laryngeal manual therapy in dysphonic women: clinical trial. *J Voice*. 2015;29(2):200-208. doi:10.1016/j.jvoice.2014.06.003<br>• Anhaia TC, da Silva Klahr P, Ourique AAB, et al. Effects of two interventions in teachers with voice complaints. *Audiol Commun Res*. 2014;19:186–193. doi:10.1590/S2317-64312014000200014.<br>• Van Lierde KM, De Bodt M, Dhaeseleer E, Wuyts F, Claeys S. The treatment of muscle tension dysphonia: a comparison of two treatment techniques by means of an objective multiparameter approach. *J Voice*. 2010;24(3):294-301. doi:10.1016/j.jvoice.2008.09.003<br>• Mathieson L, Hirani SP, Epstein R, Baken RJ, Wood G, Rubin JS. Laryngeal manual therapy: a preliminary study to examine its

	treatment effects in the management of muscle tension dysphonia. *J Voice*. 2009;23(3):353-366. doi:10.1016/j.jvoice.2007.10.002<br>• Roy N, Nissen SL, Dromey C, Sapir S. Articulatory changes in muscle tension dysphonia: evidence of vowel space expansion following manual circumlaryngeal therapy. *J Commun Disord*. 2009;42(2):124-135. doi:10.1016/j.jcomdis.2008.10.001<br>• Dromey C, Nissen SL, Roy N, Merrill RM. Articulatory changes following treatment of muscle tension dysphonia: preliminary acoustic evidence. *J Speech Lang Hear Res*. 2008;51(1):196-208. doi:10.1044/1092-4388(2008/015)<br>• Roy N, Ferguson NA. Formant frequency changes following manual circumlaryngeal therapy for functional dysphonia: Evidence of laryngeal lowering? *J Med Speech Lang Pathol*. 2001;9(3):169-75.<br>• Roy N, Bless DM, Heisey D, Ford CN. Manual circumlaryngeal therapy for functional dysphonia: an evaluation of short- and long-term treatment outcomes. *J Voice*. 1997;11(3):321-331. doi:10.1016/s0892-1997(97)80011-2<br>• Roy N, Leeper HA. Effects of the manual laryngeal musculoskeletal tension reduction technique as a treatment for functional voice disorders: perceptual and acoustic measures. *J Voice*. 1993;7(3):242-249. doi:10.1016/s0892-1997(05)80333-9
#2	Sixteen articles included eclectic treatment approaches (using additional voice therapeutic or other techniques) that included manual laryngeal therapy.<br><br>• Kruse LR, Fujiki RB. Voice Therapy for Puberphonia in the Adolescent Male: A Clinical Case Study. *Perspectives of the ASHA Special Interest Groups*. 2026;11:316-330. doi:10.1044/2025_PERSP-25-00222<br>• Burgos-Mansilla B, Schneeberger-Hitschfeld P, Astete-Barra K, Méndez-Rojas A, Ortiz-Comino L. Effects of Manual Therapy on Oral Opening, Swallow Function and Upper Quarter Mobility in Chilean Survivors of Head and Neck Cancer: A Study Protocol for a Controlled, Randomised Study (MAnual ThErapy for Oral Opening [MATEO] Study). *BMJ Open*. 2025;15(9):e097131. doi:10.1136/bmjopen-2024-097131<br>• Vahid M, Mansuri B, Farzadi F, Tohidast SA, Bagheri R, Scherer RC. Immmediate Effects of Combining Kinesio Tap with Voice Therapy in Patients with Muscle Tension Dysphonia. 2025;39(2):429-438. doi: 10.1016/j.jvoice.2022.09.006<br>• Tate AD, Tomlinson CA, Francis DO, Wishick ED, Lowery AS, Watkins MO, Stewart TG, Gong WH, Gilbert MR, Garrett CG. Physical Therapy for Muscle Tension Dysphonia with

	Cervicalgia. *Ear, Nose & Throat Journal*. 2024; 103(8):509-517. doi:10.1177/014556132110632<br>• Sarin V, Chatterjee A. Efficacy of Voice Therapy in Rehabilitation of Muscle Tension Dysphonia in Patients of Nonlaryngeal Head and Neck Cancer: A Sequelae of Chemoradiotherapy. *Indian J Otolaryngol Head Neck Surg*. 2023;75(4):3739-3749. doi:10.1007/s12070-023-04072-x<br>• Veselinović M, Škrbić R, Mumović G, Dragičević D, Despić D, Milankov V. Voice Improvement after Voice Therapy in Female Patients with Unilateral Vocal Fold Paralysis after Thyroid Surgery. *Folia Phoniatr Logop*. 2023;75(4):265-272. doi:10.1159/000529573<br>• Bonetti A, Šimić I, Živković-Ivanović T. Voice Outcomes as a Results of Voice Therapy After Lobectomy and Thyroidectomy. *Acta Clin Croat*. 2020; 59:18-24. Doi: 10.20471/acc.2020.59.s1.02<br>• DePietro JD, Rubin S, Stein DJ, Golan H, Noordzij JP. Laryngeal Manipulation for Dysphagia with Muscle Tension Dysphonia. *Dysphagia*. 2018;33(4):468-473. doi:10.1007/s00455-018-9875-x<br>• Kim JS, Lee DW, Choi CH, Choi SH. Effects of Laryngeal Massage and Semi-occluded Vocal Tract Exercises for Patients with Hyperfunctional Dysphonia. *Communication Sciences & Disorders*. 2017;22(4):806-817. doi:10.12963/csd.17434<br>• Ishige M, Omori F, Nito T, Kobayashi T, Suzuki M. Voice Therapy for a Case of Recalcitrant Mutational Dysphonia. *Japan Journal of Logopedics and Phoniatrics*. 2015;56(3):244-249. doi:10.5112/jjlp.56.244<br>• Marszałek S, Niebudek-Bogusz E, Woźnicka E, Malińska J, Golusiński W, Śliwińska-Kowalska M. Assessment of the influence of osteopathic myofascial techniques on normalization of the vocal tract functions in patients with occupational dysphonia. *Int J Occup Med Environ Health*. 2012;25(3):225-235. doi:10.2478/S13382-012-0041-7<br>• Won YS. Influence of Manual Facilitation Technique on Swallowing Disorder and Aspiration Pneumonia Caused by Sever Dysphagia with Stroke. *Journal of Physical Therapy Science*. 2012;24(9). doi:10.1589/jpts.24.909<br>• Faure MA. Phoniatric Management of 3 Clinical Cases of Voice Disorders Involving the Recurrent Nerve. *Rev Laryngol Otol Rhinol*. 2012;133(1):55-57.

	• Roch JB, Piron A, Collet-Beillon F, Benali J, Ayrault S, Moreau AC. Manual Care in Speech Rehabilitation. *Rev Largyngol Otol Rhinol (Bord).* 2005;126(5):361-364.<br>• Van Lierde K, De Ley S, Clement G, De Bodt M, Van Cauwenberge P. Outcome of Laryngeal Manual Therapy in Four Dutch Adults with Persistent Moderate-to-Severe Vocal Hyperfunction: A Pilot Study. *Journal of Voice.* 2004;18(4):467-474. doi:10.1016/j.jvoice.2004.02.003<br>• Taguchi A, Hyodo M, Kobayashi J. Voice Therapy for Adductor Spasmodic Dysphonia. *Jibi Inkoka Rinsho*. 202;95(12):1271-1275. doi:10.5631/jibirin.95.1271
#3	Three articles combined different manual approaches together in which a clear effect of a specific method cannot be assessed based on heterogenous concept/procedure of the treatment<br><br>• Kim BG, Choi SH, Lee K, Choi CH. Efficacy of Laryngeal Massage in Patients with Muscle Tension Dysphonia: Changes in Surface Electromyography (sEMG) Activity. Commun Sci Disord 2023; 28(3): 658-668. doi: 10.12963/csd.23997<br>• Dahl KL, François FA, Buckley DP, Stepp CE. Voice and Speech Changes in Transmasculine Individuals Following Circumlaryngeal Massage and Laryngeal Reposturing. *Am J Speech Lang Pathol*. 2022;31(3):1368-1382. doi:10.1044/2022_AJSLP-21-00245<br>• Buckley DP, Dahl KL, Cler GJ, Stepp CE. Transmasculine Voice Modification: A Case Study. *J Voice*. 2020;34(6):903-910. doi:10.1016/j.jvoice.2019.05.003<br>• Salehi A, Izadi F, Gholami Tehrani L, Rahgozar M. Voice-based laryngeal manual therapy in treatment of hyperfunctional voice disorders: A preliminary study. *Aud Vestib Res*. 2013;22(3):52-62.
#4	Four articles included manual and massage approaches where the larynx wasn't included or it was unclear whether the larynx was included.<br><br>• Magliano R. Massage Therapy for a Voice Student with Behavioral Dysphonia When Singing: A Case Report. *Int J Ther Massage Bodyw.* 2026;19(2):27-45. doi: 10.3822/ijtmb.v19i2.1215<br>• Andriollo DB, Frigo LF, Cielo CA. Effect of Pompage on Vocal and Respiratory Measurements from Female Teachers: A Randomized Controlled Clinical Trial. 2023;75:295-305. doi: 10.1159/000529832<br>• Cardoso R, Meneses RF, Lumini-Oliveira J, Pestana P. Myofascial Release Effects in Teachers' Posture, Muscle Tension and Voice

	Quality: A Randomized Controlled Trial. *J Voice*. 2023;37(4):635.e15-635.e27. doi:10.1016/j.jvoice.2021.03.029<br>• Liang FY, Huan XM, Chen L, Huang, YZ, Zhang XY, Su JH, Wang YJ, Yang JS, Zheng YQ, Mei XS, Guan Z. Voice Therapy Effect on Mutational Falsetto Patients: A Vocal Aerodynamic Study. *Journal of Voice*. 2017;31(1):114.e1-114.e5. doi:10.1016/j.jvoice.2016.03.017
#5	One article described insufficiently the manual approach with the result that it cannot be clearly assigned to a specific manual program.<br><br>• Steidl EMDS, Gonçalves BFDT, Rockenbach NM, Pasqualoto AS, Mancopes R. Outcomes of manual therapy on the biomechanics of swallowing in individuals with COPD. *Codas*. 2021;33(5):e20200203. doi:10.1590/2317-1782/20192020203
#6	Three articles used insufficient documentation of outcome measures.<br><br>• Kennard EJ, Lieberman J, Saaid A, Rolfe KJ. A Preliminary Comparison of Laryngeal Manipulation and Postural Treatment on Voice Quality in a Prospective Randomized Crossover Study. *J Voice*. 2015;29(6):751-754. doi:10.1016/j.jvoice.2014.09.026<br>• Awan SN, Roy N. Outcomes Measurement in Voice Disorders: Application of an Acoustic Index of Dysphonia Severity. *Journal of Speech, Language, and Hearing Research*. 2009;52:482-499. doi:1092-4388/09/5202-0482<br>• Roy N, Ford CN, Bless DM. Muscle tension dysphonia and spasmodic dysphonia: the role of manual laryngeal tension reduction in diagnosis and management. *Ann Otol Rhinol Laryngol*. 1996;105(11):851-856. doi:10.1177/000348949610501102
#7	One article included manual intervention on vocally healthy participants without a verifiable risk factor of the clinical population.<br><br>• Laukkanen AM, Leppänen K, Tyrmi J, Vilkman E. Immediate effects of 'voice massage' treatment on the speaking voice of healthy subjects. *Folia Phoniatr Logop*. 2005;57(3):163-172. doi:10.1159/000084136
#8	31 articles were reviews not including meta-analyses, or a meta-analysis containing no information about the effect of manual therapy specifically<br>• Sebastian S, Roshini VS, Thanooja CV. Evidence for Standalone Voice Therapy in the Management of Spasmodic Dysphonia: A Systematic Review and Identification of Research Gaps. *Journal of Voice*. In press. doi:10.1016/j.jvoice.2026.06.054<br>• Adabi Mohazzab FZ, Aghadoost S, Tahamtan M, Dabirmoghaddam P, Aghajanzadeh M. Voice Therapy in

Mutational Falsetto: A Narrative Review. *Journal of Voice*. In press. doi:10.1016/j.jvoice.2026.04.026

- Ruckart KW, McKone M, Phillips JG, Krekelberg MO, Sussman KA, Madden LL. Efficacy of Speech-Language Pathology Interventions for Globus Pharyngeus: A Scoping Review. *Journal of Voice*. 2026;40(4):1102-1109. doi:10.1016/j.jvoice.2024.02.027
- Garabet R, Melley LE, Sataloff RT. Noninvasive Management of Voice Disorders: An Umbrella Review. *Journal of Voice*. In press. doi:10.1016/j.jvoice.2024.03.027
- Avilés Muñoz A. Direct Voice Therapy Approaches to Treat Muscle Tension Dysphonia. *Ciencias de la Conducta/Behavioral Sciences Journal*. 2024;39(1).
- Aghadoost S, Tahamtan M, Dabirmoghaddam P, Aghajanzadeh M. A Narrative Review on Laryngeal Manual Therapy Methods in Muscle Tension Dysphonia. *Indian Journal of Otolaryngology and Head & Neck Surgery*. Published online July 18, 2024. doi:10.1007/s12070-024-04896-1
- Flock L, King SR. A Manual Therapy in Need of a Manual: Conceptualizing and Comparing Existing Vocal Massage Protocols and Their Use in the Treatment of Voice Problems. *Voice and Speech Review*. 2024;18(3). doi: 10.1080/23268263.2023.2262181
- Cheng TW, Chu EH, Lin CH, Tseng SH, Hou WH, Lai CH. Effectiveness of Voice Therapy on Voice Quality and Function in Individuals with Unilateral Vocal Fold Paralysis: Systematic Review and Meta-Analysis. *Journal of Voice*. In press. doi:10.1016/j.jvoice.2024.07.006
- Fritz W. The Mechanism of Action for Laryngeal Manual Therapies: The Need for an Update. *Current Opinion in Otolaryngology & Head and Neck Surgery*. 2024;32(3):151-155. doi:10.1097/MOO.0000000000000966
- Toles LE, Harris AL. Direct and Indirect Voice Therapy Approaches for Unloading the Laryngeal Mechanism in Professional Voice Users. *Current Otorhinolaryngology Reports*. 2023;11(3):318-324. doi:10.1007/s40136-023-00483-0
- Lester-Smith RA, Miller CH, Cherney LR. Behavioral Therapy for Tremor or Dystonia Affecting Voice in Speakers with Hyperkinetic Dysarthria: A Systematic Review. *Journal of Voice*. 2023;37(4):P561-573. doi:10.1016/j.jvoice.2021.03.026
- Barsties v. Latoszek B, Watts CR, Hetjens S, Neumann K. The Efficacy of Different Voice Treatment for Vocal Fold Polyps: A Systematic Review and Meta-Analysis. *Journal of Clinical Medecine*. 2023;12:3451. doi: /10.3390/jcm12103451
- Jovanović SS, Arsić SD, Stojanović Jovanović BN, Kljajić DM, Trajkov MT. Application of Manual Therapy in Muscle Tensional Dysphonia. *PONS Medical Journal (PONS Medicinski Časopis)*. 2018;15(4):129-137. doi:10.5937/pomc18-27228

- Pereira GdC, Lemos IdO, Gadenz CD, Cassol M. Effects of Voice Therapy on Muscle Tension Dysphonia: A Systematic Literature Review. *Journal of Voice.* 2018;32(5):546-552. doi: 10.1016/j.jvoice.2017.06.015
- Carding P, Bos-Clark M, Gillivan-Murphy P, Jones SM, Walton C. Evaluating the Efficacy of Voice Therapy for Functional, Organic and Neurological Voice Disorders. *Clinical Otolaryngology.* 2016;42:201-2017. doi: 10.1111/coa.12765
- Beuhausen U. Stimmtherapeutische Methoden – Zwischen Tradition und Evidenzbasierung. *Forum Logopädie*. 2013;5(27):34-39.
- Ongkasuwan J, Friedman EM. Is Voice Therapy Effective in the Management of Vocal Fold Nodules in Children? *The Laryngoscope*. 2013;123(12):2930-2931. doi:10.1002/lary.23830
- Lehnert B. On Dysphonia, the Cervical Spine and Manual Therapies. *Sprache Stimme Gehör.* 2011;35:e144-e148. doi: 10.1055/s-0031-1297980
- Mathieson L. The Evidence for Laryngeal Manual Therapies in the Treatment of Muscle Tension Dysphonia. *Current Opinion in Otolaryngology & Head and Neck Surgery*. 2011;19(3):171-176. doi:10.1097/MOO.0b013e3283448f6c
- Van Houtte E, Van Lierde K, Claeys S. Pathophysiology and Treatment of Muscle Tension Dysphonia: A Review of the Current Knowledge. *Journal of Voice*. 2011;25(2):202-207. doi:10.1016/j.jvoice.2009.10.009
- Schönweiler R. Dysphonie – Manualtherapie: Alternative zur Übungstherapie. *Sprache Stimme Gehör.* 2011;35(1):3-4. doi: 10.1055/s-0031-1276667
- Rosing-Schow A, Pedersen SG. VoiceRelease. *Logopedics Phoniatrics Vocology.* 2010;35(4):166-168. doi: 10.3109/14015431003599004
- Roy N. Assessment and Treatment of Musculoskeletal Tension in Hyperfunctional Voice Disorders. *International Journal of Speech-Language Pathology*. 2008;10(4):195-209. doi:10.1080/17549500701885577
- Deeter A. Understanding Massage: How Massage Therapy Can Enhance Self-Care and Vocal Health. *Journal of Singing.* 2006;62(5):541-545.
- Emerich K. Nontraditional tools helpful in the treatment of certain types of voice disturbances *Current Opinion in Otolaryngology & Head & Neck Surgery*. 2003;11(3):149-153.
- Piron A. La Manipulation du Larynx. *Rev Laryngol Otol Rhinol.* 2001 ;122(5) :357-360.
- Rubin JS, Lieberman J, Harris TM. Laryngeal Manipulation. *Otolaryngologic Clinics of North America*. 2000;33(5):1017-1034. doi:10.1016/S0030-6665(05)70261-9

	• Roy N, Bless DM. Manual Circumlaryngeal Techniques in the Assessment and Treatment of Voice Disorders. *Current Opinion in Otolaryngology and Head and Neck Surgery.* 1998;6(3):151-155.<br>• de la Bretèque A. Rehabilitation of Disorders of the Breaking of Voice. *Rev Laryngol Otol Rhinol (Bord).* 1995;116(4):271-272.<br>• Chen P. Massage for the Treatment of Voice Ailments. *J Tradit Chin Med.* 1991;11(3):209-215.<br>• Marvaud J. Relaxation in Voice Pathology. *Rev Laryngol Otol Rhinol (Bord).* 1985;106(4):299-300.
#9	6 articles were non-journal articles, for example conference abstracts or thesis publications.<br>• Tawiah G, Rayner L. The Role of Physiotherapy as Part of a Multidisciplinary Team Approach to Vocal Rehabilitation and Care of the Professional Voice. *Physiotherapy*. 2025;126(Suppl 1):101497. doi:S0031-9406(25)00035-5<br>• François FA. The Effect of Laryngeal Manual Therapy and Laryngeal Reposturing with Voicing on Fundamental Frequency and Estimated Vocal Tract Length in Transmasculine Speakers [Master thesis]. Boston (MA): Boston University; 2015.<br>• Enver N. Mutational Falsetto (Puberophony) and its Treatment. *Psychiatry and Clinical Psychopharmacology.* 2018;28:373.<br>• Lewin J, Woodall H, Porsche C, Barrow M, Hutcheson K. Manual Therapy: Integration into a Speech and Swallowing Rehabilitation Program for Head and Neck Cancer. *Archives of Physical Medecine & Rehabilitation.* 2017;98(10):E3. doi: S0003-9993(17)30555-5<br>• Depietro JD, Stein DJ, Rippberger MJ, Noordzij JP. Laryngeal Manipulation for Dysphagia from Muscle Tension Dysphonia. *Otolaryngology – Head and Neck Surgery.* 2014;151(1):P190. doi:10.1177/0194599814541629a164<br>• Del Pozo Recio LT. Immediate Acoustic Effects of Laryngeal Manual Therapy in a Group of Patients with Muscle Tension Dysphonia Using the Laryngograph [Master Thesis]. London, United Kingdom: University College London; 2006.
